# The Effect of Neoadjuvant Chemotherapy on Lymph Node Metastasis of FIGO Stage IB1-IIB Cervical Cancer: A Systematic Review and Meta-Analysis

**DOI:** 10.3389/fonc.2020.570258

**Published:** 2020-11-05

**Authors:** Bingxin Chen, Liming Wang, Ci Ren, Hui Shen, Wencheng Ding, Da Zhu, Lu Mao, Hui Wang

**Affiliations:** Department of Obstetrics and Gynecology, Tongji Hospital, Tongji Medical College, Huazhong University of Science and Technology, Wuhan, China

**Keywords:** uterine cervical neoplasms, neoadjuvant chemotherapy, surgery, lymphatic metastasis, lymph node metastasis

## Abstract

**Objectives:**

This study aimed to assess the effect of neoadjuvant chemotherapy (NACT) on the rate of lymph node metastasis (LNM) in FIGO stage IB1-IIB cervical cancer patients and compare the LNM between NACT plus surgery and surgery only.

**Methods:**

We identified 34 eligible studies in PubMed, Web of Science, Cochrane Library, and EMBASE from inception to July 27, 2019. Data analyses were performed by Stata (version 13) and Revman (version 5.3).

**Results:**

In these 34 included studies, the pooled incidence of LNM was estimated as 23% (95% CI, 0.20-0.26; I^2^ = 79.6%, *P*<0.001). In the subgroup analysis, we identified five factors, including study type, year of publication, continents from which patients came, histological type and the FIGO stage. When taking FIGO stage into consideration, the LNM rate was 13% in stage IB (95% CI: 0.10-0.15; I^2^ = 5.5%, *P*=0.385), 23% in stage IIA (95% CI: 0.18-0.28; I^2^ = 0%, *P*=0.622), and 27% in stage IIB (95% CI: 0.20-0.33; I^2^ = 0%, *P*=0.898), respectively. Through the comparison between NACT plus surgery and surgery only based on the six randomized controlled trials, the incidence of positive lymph nodes was lower in patients receiving NACT plus surgery than surgery only (RR=0.57, 95% CI: 0.39-0.83; I^2^ = 60.5%, *P*=0.027). The 5-year OS was higher in the NACT + surgery group than surgery-only group (RR=1.13, 95% CI: 1.03-1.23; I^2^ = 0.0%, *P*=0.842).

**Conclusions:**

Among cervical cancer in stage IB1-IIB, the preoperative NACT plus radical surgery resulted in a 23% probability of LNM, which was lower than those receiving radical surgery only. In stage IIA and IIB, the effect of NACT to reduce LNM was more obvious.

## Introduction

Despite the development of comprehensive treatment technology, the cases of cervical cancer have increased from 528,000 in 2012 to 570,000 in 2018, and the deaths have increased from 266,000 in 2012 to 311,000 in 2018 (1, 2). The higher regional morbidity incidence was found in developing countries, which was 3 to 10 times higher than developed areas (1, 3, 4). Therefore, cervical cancer has become one of the most important public health challenges. According to the National Comprehensive Cancer Network (NCCN) guidelines, it is recommended that patients with stage IB1 and IIB cervical cancer undergo radical hysterectomy (RH) and/or chemoradiation (5). However, the traditional treatment methods would seriously affect patients’ endocrine and reproductive function, and some patients lost the chance to get effective treatment when diagnosed due to the large tumor size (3, 4). Gradually, preoperative neoadjuvant chemotherapy (NACT) caught clinicians’ attention, which might provide more survival benefits (3–9).

Since neoadjuvant chemotherapy was proposed and applied to the treatment of cervical cancer in the 1980s, more and more studies have focused on NACT (7–14). NACT can treat patients with distant metastases and shows great efficacy in both reducing recurrence and improving survival (7–9, 12) In 2019, the NCCN pointed out that select patients with FIGO stage IB2-IIB disease may accept RH or NACT followed by RH (5).

Actually, there existed many risk factors affecting the prognosis of cervical cancer, among which lymph node metastasis (LNM) was one of the most important high risk factors (3, 4, 6, 8, 9). The presence of LNM and the increase in positive lymph node (LN) number were followed with higher recurrence rates and lower survival rates (15). Therefore, to explain the efficacy of NACT, we need to pay attention to the impact of NACT on LNs, which the previous studies ignored.

Therefore, the aim of this study was to conduct a meta-analysis of the literature on NACT followed by radical surgery and to evaluate the effect of NACT on LNM in FIGO stage IB1-IIB cervical cancer.

## Methods

### Search Strategy

This study was conducted on the basis of the preferred reporting items for systematic reviews and meta-analysis (PRISMA) statement and registered in Prospero (PROSPERO CRD42018117658) (16, 17). We searched PubMed, Web of Science, Cochrane Library, and EMBASE for relative studies. In order to search the PubMed database, we used the following combination of terms: (Uterine Cervical Neoplasms[MeSH Terms] OR ((cervix*[Title/Abstract] OR cervical*[Title/Abstract] OR uterine cervix*[Title/Abstract] OR cervix uteri*[Title/Abstract]) AND (cancer*[Title/Abstract] OR tumor*[Title/Abstract] OR tumour*[Title/Abstract] OR neoplas*[Title/Abstract] OR carcinoma*[Title/Abstract] OR malignanc*[Title/Abstract] OR carcinogenesis*[Title/Abstract] OR intraepithelial neoplas*[Title/Abstract]))) AND (Lymphatic Metastasis[MeSH Terms] OR ((lymph node* [Title/Abstract] OR nodal[Title/Abstract] OR node*[Title/Abstract] OR lymphatic[Title/Abstract]) AND (metastasis [Title/Abstract] OR recurrence [Title/Abstract] OR invasion [Title/Abstract] OR Metastatic Ratio[Title/Abstract]))) AND ((neoadjuvant[Title/Abstract] OR preoperat*[Title/Abstract] OR upfront[Title/Abstract] OR primary[Title/Abstract] OR induction[Title/Abstract] OR adjuvant[Title/Abstract]) AND (chemotherapy[Title/Abstract] OR treatment [Title/Abstract] OR therapy[Title/Abstract])). We used an appropriately modified PubMed search strategy to search the other three databases, including Web of Science, Cochrane Library, and EMBASE. The detailed search strategy is shown in **Supplementary Appendix 1**. The year of publication is limited to the period from inception to July 27, 2019. We also searched the publications that cited those included articles and other related articles.

### Inclusion and Exclusion Criteria

The eligible studies must meet the inclusion criteria as follows: (i) the patient was pathologically diagnosed as stage IB1-IIB cervical cancer; (ii) NACT was platinum based; (iii) the surgery was extensive RH; (iv) the study provided complete data, especially LN status.

Studies were excluded if they met any of these criteria: (i) The studies reported patients with other malignant diseases; (ii) the studies included patients receiving other treatments in addition to NACT and radical surgery; (iii) non-English literature; and (iv) reviews, meta-analysis, case reports, conference articles, and articles without clear data.

Two independent researchers (BC and LM) filtered all publications. Disagreements were determined by group discussion with a third researcher (HW).

### Data Extraction and Outcomes

Data were extracted by two independent researchers (BC and LM) and filled in standardized data-collection forms (**Supplementary Table 1**). Opposing opinions were solved by discussion with a third researcher (HW). If the original article did not report detailed data, the researchers would contact the first author by e-mail. Extracted data included authors, country, continent of patients, study type, year of publication, number of patients, age of patients, FIGO stage, histological type, NACT regimen, NACT cycle, and LNM. The endpoint was LNM. The rate of LNM was defined as the ratio of observed number of patients with positive LNs divided by the number of total patients undergoing treatment.

### Assessment of Risk of Bias

The quality of each identified study was assessed according to a modified version based on the Cochrane Collaboration’s risk of bias tool (**Supplementary Tables 2, 3**). We took the following five items as criteria: assessment of record, assessment of diagnosis, assessment of LN status, assessment of loss to follow-up, selective inclusion, and exclusion. In each criterion, the study was evaluated as low, unclear, and high risk of bias. The studies were classified as low risk of bias only if the five criteria were all at low risk of bias. The risk of bias was examined by two reviewers (BC and LM) independently, and discrepancies were resolved by consensus. If required, a third reviewer (HW) would join them.

### Statistical Analysis

Meta analyses were conducted through Stata (version 13) and Revman (version 5.3). We estimated the LNM rate with Freeman-Turkey double arcsine transformation because there were a large proportion of data that were close to the margins of the possible interval (0% or 100%) (18). Relative risk (RR) was used to compare the LNM rates between two treatment groups. The heterogeneity between the individual studies was quantitatively estimated with the Chi-square and I^2^ statistics (19, 20). When *P* was greater than 0.1, it was considered to be no heterogeneity; otherwise, there existed significant heterogeneity. I^2^ was used to further measure heterogeneity because of the limits of the Chi-square statistic. A threshold of I^2^ below 50% indicated no significant heterogeneity, and I^2^ over 50% suggested high heterogeneity. Fixed effects models were considered when there was no between-study heterogeneity. In contrast, we used the random effects model. Sensitivity and subgroup analyses were conducted to evaluate the effect of various variables on outcomes. In order to detect potential publication bias, we performed the visual inspection of funnel plot and Egger’s test (21, 22). All *p* values are two-sided, and a *p* value less than 0.05 was considered as statistically significant.

## Results

### Characteristics of Studies

**Figure 1:**
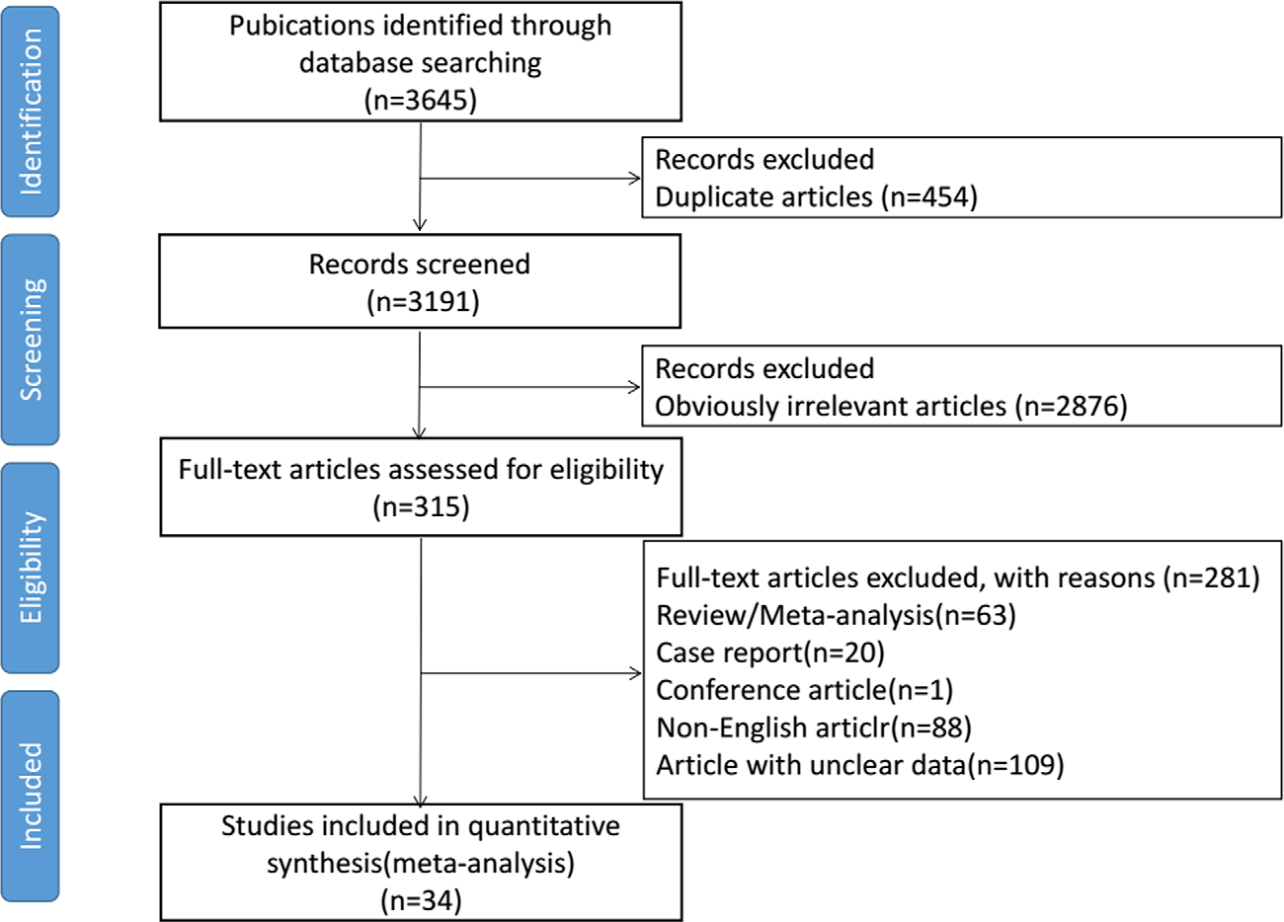
Flow diagram of literature selection process for the meta-analysis.

Initially, we identified 3645 eligible studies. After screening, 34 studies were finally included in this meta-analysis, consisting of 3813 patients (6–8, 12–14, 23–50). The selection process is shown in **Figure 1**. The detailed characteristics for each included article are systematically summarized in **Supplementary Table 1**. Eight studies were randomized controlled trials (RCTs) with matched data in the experimental arm (6, 13, 14, 24, 32, 41, 45, 47), three were prospective cohorts (23, 40, 49), and the other 23 studies were retrospective cohorts (7, 8, 12, 25–31, 33–39, 42–44, 46, 48, 50). The largest study included 705 women (8), and the smallest included 20 women (42). 73.5% of studies (n=25) contained more than 50 patients. In 61.7% of the studies (n=21), the preoperative chemotherapy cycle was 2–3 weeks. The postoperative adjuvant radiotherapy or chemotherapy was determined by the patient’s surgical outcome and discretion of their clinicians. Through the assessment of risk of bias, 10 of the 34 studies did not meet the requirements for low bias risk (13, 14, 23, 25, 27, 32, 40, 42, 45, 49). In these 10 studies, five were due to loss of follow-up and four were not at low risk in assessment of LN status. One study reported the record of 51 patients without a clear source (**Supplementary Table 3**).

### Summary LNM Rate

**Figure 2:**
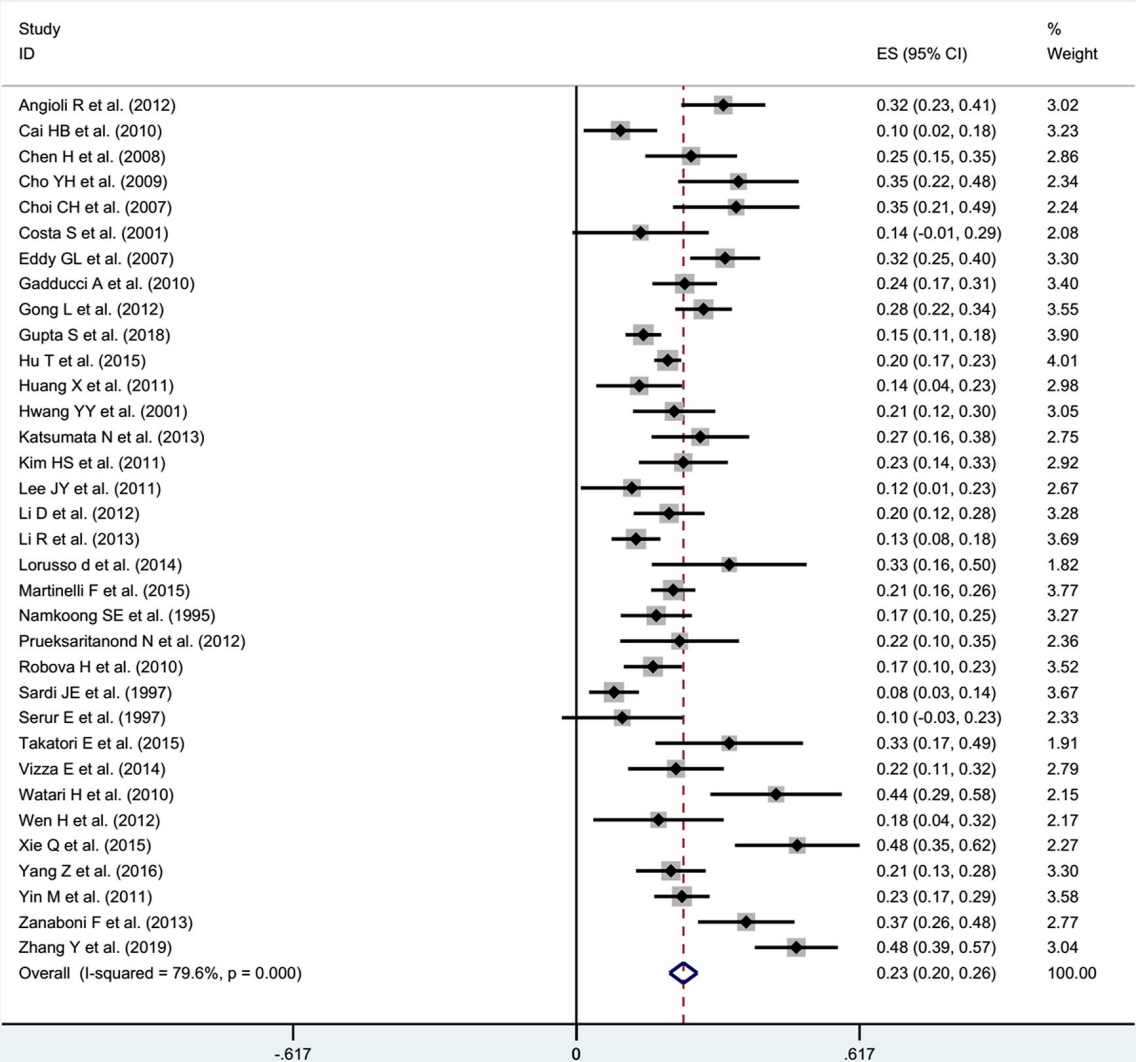
Forest plot of the estimated LNM rate in cervical cancer patients receiving NACT plus RH.

**Figure 3:**
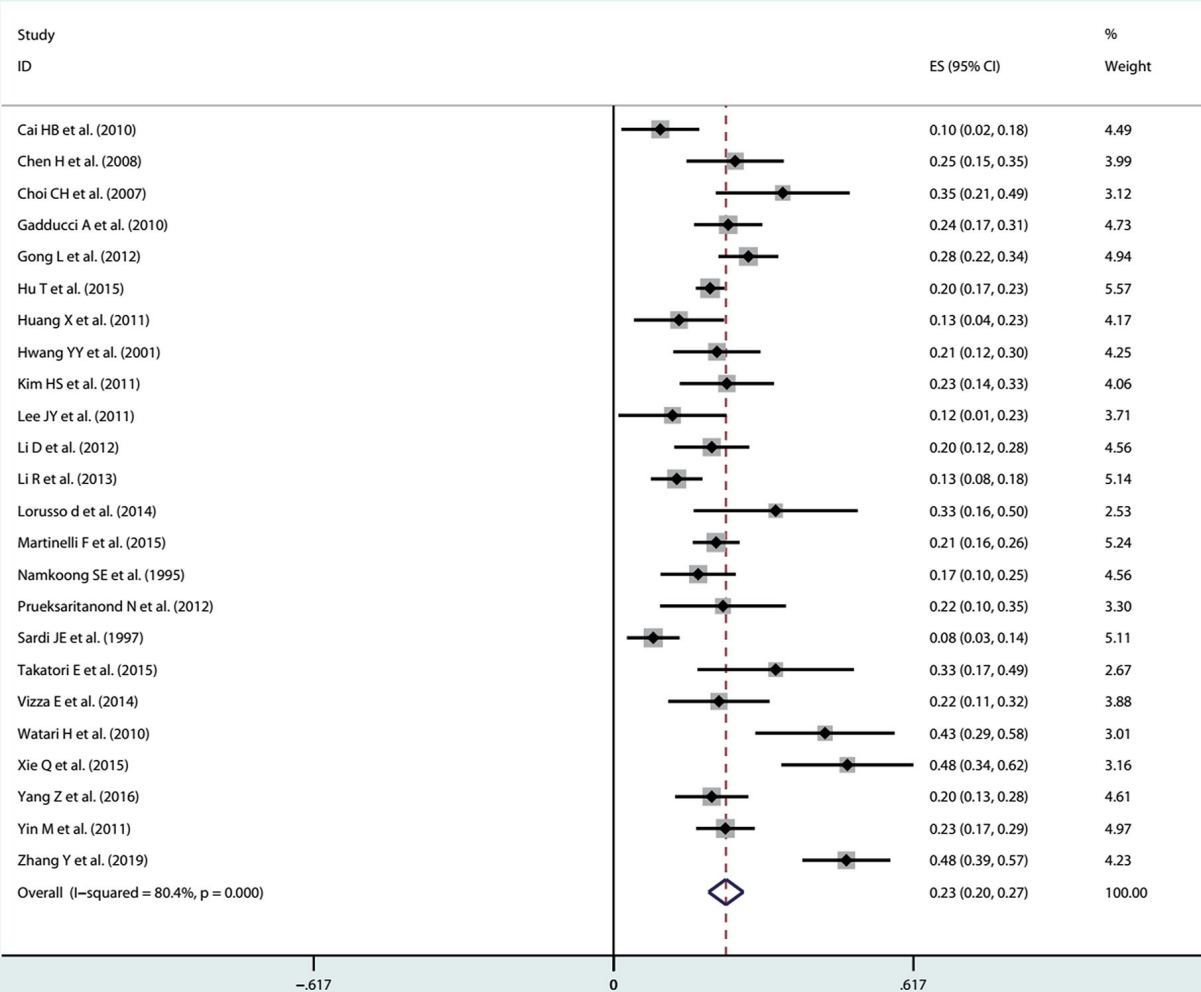
After removing the literature with high risk of bias, the forest plot of estimated LNM rate.

After receiving NACT in patients with stage IB1-IIB cervical cancer, the pooled incidence was 23% (95% CI: 0.20-0.26) with significant heterogeneity (I^2^ = 79.6%, *P*<0.001) in the random effects model (**Figure 2**). Through Freeman-Turkey double arcsine transformation, the transformed estimate of LNM rate was 23.8% (95% CI: 0.209-0.269), which mostly matched the overall random effect estimates (**Supplementary Table 4**). Further, we removed the literature with high risk of bias and estimated the LNM rate to be 23% (95% CI: 0.20-0.27; I^2^ = 80.4%, *P*<0.001) (**Figure 3**).

### Subgroup Analysis

We took the following factors into consideration for the subgroup analysis: study types, the year of publication, patients’ continents, histological types, and FIGO stage (**Table 1**). **Supplementary Figures 4A**, **B** shows the rate of positive LNs in subgroups defined by the year of publication and FIGO stage. From 1995 to 2009, LNM rates showed an upward trend (1995-1999: 12%, 95% CI: 0.05-0.18; 2000-2004: 19%, 95% CI: 0.12-0.27; 2005-2009: 31%, 95% CI: 0.26-0.36). From 2010 to present, LNM rates fluctuated (2010-2014: 22%, 95% CI: 0.19-0.26; 2015-present: 28%, 95% CI: 0.20-0.35). **Supplementary Figure 4B** shows that the LNM rate was 13% (95% CI: 0.10-0.15) in stage IB, 23% (95% CI: 0.18-0.28) in stage IIA, and 27% (95% CI: 0.20-0.33) in stage IIB.

**Table 1:** Subgroup analysis of the 34 studies.

Subgroups	No. of trials	Rates^1^	95% CI	No. of patients	Heterogeneity
study types					
retrospective study	23	24%	(0.21-0-28)	887	I^2^ = 76.6%, *P*=0.000
prospective study	3	28%	(0.15-0.41)	313	I^2^ = 85.5%,*P*=0.001
randomized controlled study	8	19%	(0.13-0.25)	2613	I^2^ = 81.2%,*P*=0.000
Total	34	23%	(0.20-0.26)	3813	I^2^ = 79.6%,*P*=0.000
year of publication					
1995-1999	3	12%	(0.05-0.18)	210	I^2^ = 45.4%,*P*=0.160
2000-2004	2	19%	(0.12-0.27)	101	I^2^ = 0.0%,*P*=0.409
2005-2009	4	31%	(0.26-0.36)	314	I^2^ = 0.0%,*P*=0.530
2010-2014	18	22%	(0.19-0.26)	1581	I^2^ = 69.7%,*P*=0.000
2015-present	7	28%	(0.20-0.35)	1607	I^2^ = 90.4%,*P*=0.000
Total	34	23%	(0.20-0.26)	3813	I^2^ = 79.6%,*P*=0.000
continents of patients^2^					
Europe	8	24%	(0.19-0.29)	839	I^2^ = 60.7%,*P*=0.013
Asia	23	24%	(0.20-0.27)	2711	I^2^ = 79.9%,*P*=0.000
South America and North America	2	8%	(0.03-0.13)	118	I^2^ = 0.0%,*P*=0.804
Total	33	23%	(0.20-0.26)	3668	I^2^ = 79%,*P*=0.000
histological types					
squamous cervical cancer	5	18%	(0.10-0.25)	878	I^2^ = 79.3%,*P*=0.001
non-squamous cervical cancer	3	16%	(0.03-0.29)	112	I^2^ = 69.7%,*P*=0.037
Total	6	17%	(0.11-0.23)	990	I^2^ = 74.7%,*P*=0.000
FIGO stage^3^					
IB	7	13%	(0.10-0.15)	699	I^2^ = 5.5%,*P*=0.385
IIA	3	23%	(0.18-0.28)	308	I^2^ = 0.0%,*P*=0.622
IIB	3	27%	(0.20-0.33)	184	I^2^ = 0.0%,*P*=0.898
Total	7	17%	(0.13-0.21)	1191	I^2^ = 62.8%,*P*=0.001

^1^The relevant positive LN rates in subgroup or in total.

^2^The study by Eddy GL et al. was not included in the subgroup analysis of continents, because this study consisted of 62% were white,13% were black,19% were Hispanic,4% were Asian/Pacific islander and 1% were Other.

^3^The patients’ stages were determined according to the FIGO stage criteria at the time of diagnosis.

### Sensitivity Analysis and Publication Bias

The sensitivity analyses were performed by excluding one study at a time and did not noticeably affect the results (**Supplementary Figure 1**). The visual inspection of funnel plots and the Egger’s test showed no evidence of the presence of small study effects (**Supplementary Figure 2, 3**).

### NACT Plus Surgery Versus Surgery

**Figure 4:**
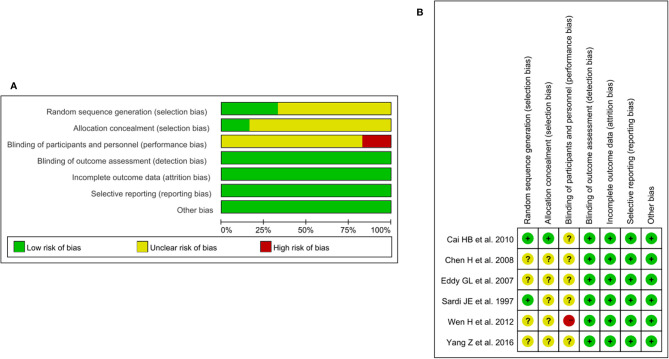
**(A)** Risk of bias graph for six RCTs. **(B)** Risk of bias summary for six RCTs.

**Figure 5:**
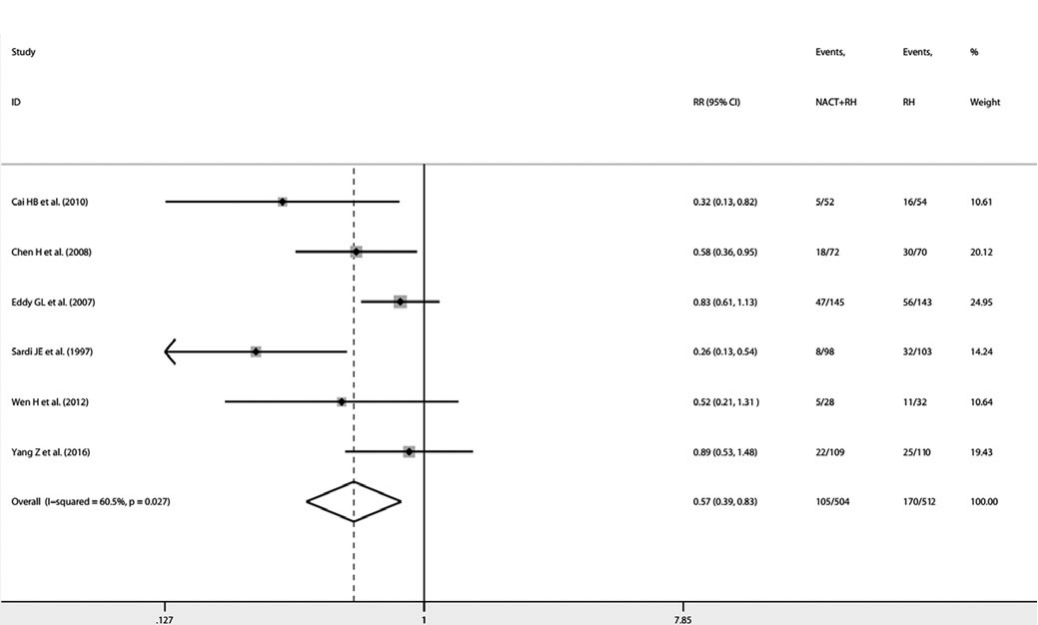
Forest plots for the LNM rate in the comparison between NACT plus RH and RH.

From the 34 included studies, we identified six two-arm RCTs including 1016 patients (6, 13, 24, 41, 45, 47). The characteristics for the six RCTs are summarized in **Table 2**. Revman (version 5.3) was used to conduct the assessment of the risk of bias for each RCT on the basis of the Cochrane Collaboration tool (51). The results are shown in **Figures 4A**, **B**. Because I^2^ = 60.5%, *P*=0.027, we used the random effects model. The pooled RR of 0.57 (95% CI=0.39-0.83) suggests a significant lower risk of LNM in the NACT plus RH group than RH group (**Figure 5**). Furthermore, after extracting the 5-year OS in 5 RCTs, the RR of OS was estimated as 1.13 (95% CI: 1.03-1.23; I^2^ = 0.0%, *P*=0.842), which suggests that the 5-year OS of the NACT+RH group was higher than that of the RH group (**Supplementary Table 5, Supplementary Figure 5**).

**Table 2:** Characteristics of the 6 RCTs in the meta-analysis.

Author	Country	Continent of patients	Year of publication	Nos. of total patients	Age (medium, range) or (mean, SD)	Patients in NACT+surgery group		Patients in surgery group	
						Nos. of LNM	Nos. of Total	Nos. of LNM	Nos. of Total
Cai et al. (24)	China	Asia	2010	106	45.6 ± 22.4	5	52	16	54
Chen et al. (6)	China	Asia	2008	142	44(25-74)	18	72	30	70
Eddy et al. (13)	USA	8	2007	288	≤30:14%,31-40:31%,41-50:32%,51-60:15%,≥61:7%	47	145	56	143
Sardi et al. (41)	Argentina	South America and North America	1997	201	38.5(24-63)	8	98	32	103
Wen et al. (45)	China	Asia	2012	60	44.53 ± 9.10	5	28	11	32
Yang et al. (47)	China	Asia	2016	219	47(23-66)	22	109	25	110

## Discussion

LNM is one of the most important risk factors affecting the prognosis of cervical cancer, so we explored the impact of NACT on LNM rates. In FIGO stage IB1-IIB cervical cancer, the positive LN rate of NACT followed by RH was 23% (95% CI: 0.20-0.26). The NACT plus RH brings more benefits in reducing LNM among the stage IIA and IIB patients. Through the comparison between NACT plus RH and RH, we further confirmed that the incidence of nodal metastasis was lower in the NACT plus RH group (RR=0.57, 95% CI: 0.39-0.83, I^2^ = 60.5%, *P*=0.027).

Based on this research, LNM occurs in 23% of patients with stage IB1-IIB cervical cancer after NACT plus RH (95% CI: 0.20-0.26, I^2^ = 79.6%, *P*<0.001), which was close to those earlier analyses (52–55). Our systematic review and meta-analysis included 34 studies, consisting of 3813 patients. Such a large scale can increase the applicability of our findings. Furthermore, the subsequent data processing and analysis, such as Freeman-Turkey double arcsine transformation and subgroup analysis, could ensure the accuracy of the findings.

The incidence of positive LNs from 1995 to 2019 show an upward trend through the subgroup analysis by the year of publication. The reason for the difference over time was speculated to be stage migration and development of diagnostic techniques. With the improvement of imaging technology and surgical methods, the accuracy and sensitivity of finding positive LNs were greatly improved. Similarly, a study based on squamous cell carcinoma of the anus noted an increase in observed positive LN rate over time, which was summarized as the Will Rogers phenomenon (56, 57).

If we divide patients into three subgroups as IB, IIA, and IIB, the between-study heterogeneity is not significant. The estimated incidence was 13% (95% CI: 0.10-0.15) in stage IB, 23% (95% CI: 0.18-0.28) in stage IIA, and 27% (95% CI: 0.20- 0.33) in stage IIB. According to the previous literature including patients receiving surgery only, the nodal metastasis rates of the FIGO stage IB, IIA, and IIB were 11.5-22%, 26.7-33%, and 39.2%-63%, respectively (52–55). Therefore, NACT plus RH was more effective in reducing LNM for patients with stage IIA and IIB.

When we consider the LNM rate in patients undergoing preoperative chemotherapy plus RH and RH only, the pooled RR of 0.57 (95% CI: 0.39-0.83, I^2^ = 60.5%, *P*=0.027) revealed the lower risk of LNM in the NACT + RH group than the RH group. When considering 5-year OS as the tumor outcome, the pooled RR of 1.13 (95% CI: 1.03-1.23; I^2^ = 0.0%, *P*=0.842) suggests a higher 5-year OS of the NACT + RH group compared with the RH group. Actually, the beneficial effects of NACT have been mentioned in many other studies. Napolitano U et al. compared the OS and disease-free survival (DFS) between the NACT group and conventional surgery or radiotherapy alone groups and found that NACT plus RH can enable more patients to undergo surgical treatment and improve OS and DFS (58). Also, Rydzewska L et al. conducted a series of related studies on NACT plus surgery for cervical cancer. In 2010, he analyzed pathological response and found a significant decrease in LNM in the NACT plus surgery group compared with the surgery group (OR: 0.54, 95% CI: 0.39-0.73; heterogeneity: *P* ≤ 0.001) (59). In 2012, Rydzewska L et al. conducted further research and got a similar significant decrease in LNM (OR: 0.54, 95% CI: 0.40-0.73; heterogeneity: *P* ≤ 0.001) (60).

However, some studies propose different perspectives. Gong L et al. provided a phase III trial based on locally advanced cervical cancer (LACC) patients to detect the incidence of nodal metastasis. Their analysis showed that the proportion of positive-node patients in the NACT plus RH group (27.8%) was similar to that in the RH group (28.8%) (61). From these data, Gong L et al. concluded that NACT plus RH did not show a significant advantage compared with RH only. However, according to the clinical features, patients in the NACT plus RH group had a higher incidence of anemia before treatment (36% vs. 16%, *p* < 0.001), larger diameter of tumor (5.5 vs. 4.9 cm, *p* < 0.001), and a higher proportion in advanced stage, especially stage IIB (57% vs. 11%, *p* < 0.001), compared with the RH group. Therefore, the conclusion lacked strong evidence.

Recently, an RCT by Gupta S et al. caught great attention (14). Through comparing the efficacy of NACT plus RH versus concomitant chemotherapy and radiotherapy (CTRT) in LACC patients, they found no advantage in pelvic positive LN rate (14.6% vs 14.2%) (14). However, their assessment of LN status was limited to the pelvis and was not based on surgical outcomes, so the results did not accurately reflect the actual status of LNs. What is more, among the included patients with different disease stages, their analysis did not fully answer the questions for patients with operable cervical cancer (stages IB2 and IIA).

There also existed several limitations. First, to limit heterogeneity, subgroup analysis was performed based on six terms. Some subgroup analysis might be limited to only 3 studies with small sample sizes. Second, several important factors, such as depth of invasion and lymph-vascular space invasion (LVSI), can significantly affect the prognosis of patients with cervical cancer, but these included articles did not provide the incidence of LNM in patients grouped by depth of invasion or LVSI. Therefore, the subgroup analysis to explore the impact of infiltration depth and LVSI was not performed. Third, some studies used different evaluation methods. Four only provided the status of LNs by preoperative MRI. Another 30 studies determined the status of both para-aortic LNs and pelvis LNs based on surgical findings. When only including the studies reporting LNM based on surgical findings, the analysis demonstrated similar results (**Supplementary Figure 6**).

## Conclusion

The rate of positive LN in stage IB1-IIB cervical cancer patients was 23% after receiving NACT plus RH. The LNM rate in the NACT plus RH group was lower than the RH group. NACT plus RH showed the more obvious effect of eliminating positive LNs in patients with stage IIA and IIB compared with previously reported surgical patients. Therefore, NACT can be considered as a valuable and reasonable treatment option in patients with stage IB1-IIB cervical cancer.

## Data Availability Statement

All datasets presented in this study are included in the article/**Supplementary Material**.

## Author Contributions

BC, LW, CR, HS, WD, DZ, LM, and HW contributed to the conception and design of the study. BC, LM, and HW organized the databases and provided methodological supports. BC and LM performed the statistical analysis. BC wrote the draft of the manuscript. LM and HW contributed to the supervision of the study. All authors critically reviewed and revised the manuscript for important intellectual content and approved the final version of the manuscript before submission. All authors contributed to the article and approved the submitted version.

## Funding

This work was supported by a key program from the National Natural Science Foundation of China (No.81830074).

## Conflict of Interest

The authors declare that the research was conducted in the absence of any commercial or financial relationships that could be construed as a potential conflict of interest.
