# Supplementary material for: The Effect of Neoadjuvant Chemotherapy on Lymph Node Metastasis of FIGO Stage IB1-IIB Cervical Cancer: A Systematic Review and Meta-Analysis
Source: Front Oncol. 2020 Nov 5;10:570258. doi: 10.3389/fonc.2020.570258 (PMC7675063; doi:10.3389/fonc.2020.570258)
Supplement: Supplementary file 1 [file DataSheet_1.docx]

Supplementary Appendix 1. Search Terms

Supplementary Table 1. Characteristics of the studies in the meta-analysis.

Supplementary Table 2. The definition of risk of bias.

Supplementary Table 3. The assessment of risk of bias.

Supplementary Table 4. Freeman-Tukey double arcsine transformation.

Supplementary Table 5. Characteristics of the 5 RCTs reporting the 5-year OS in the meta-analysis.

Supplementary Fig. 1. The sensitivity analysis

Supplementary Fig. 2. The visual inspection of funnel plots

Supplementary Fig. 3. The Egger’s test

Supplementary Fig. 4A Forest plot of LNM rate in subgroups defined by the year of publication

Supplementary Fig. 4B Forest plot of LNM rate in subgroups defined by the year of FIGO stage

Supplementary Fig. 5. Forest plots for the 5-year OS in the comparison between NACT plus RH and RH.

Supplementary Fig. 6. Forest plot of LNM rate in the 30 studies that determined lymph nodes by histological biopsy

Appendix 1.

**PubMed:**

1,Uterine Cervical Neoplasms[MeSH Terms]

2,(cervix*[Title/Abstract] OR cervical*[Title/Abstract] OR uterine cervix*[Title/Abstract] OR cervix uteri*[Title/Abstract])

3,(cancer*[Title/Abstract] OR tumor*[Title/Abstract] OR tumour*[Title/Abstract] OR neoplas*[Title/Abstract] OR carcinoma*[Title/Abstract] OR malignanc*[Title/Abstract] OR carcinogenesis*[Title/Abstract] OR intraepithelial neoplas*[Title/Abstract])

4,2 AND 3

5,1 OR 4

6,Lymphatic Metastasis[MeSH Terms]

7,(lymph node* [Title/Abstract] OR nodal[Title/Abstract] OR node*[Title/Abstract] OR lymphatic[Title/Abstract])

8,(metastasis [Title/Abstract] OR recurrence [Title/Abstract] OR invasion [Title/Abstract] OR Metastatic Ratio[Title/Abstract])

9,7 AND 8

10,6 OR 9

11, (neoadjuvant[Title/Abstract] OR preoperat*[Title/Abstract] OR upfront[Title/Abstract] OR primary[Title/Abstract] OR induction[Title/Abstract] OR adjuvant[Title/Abstract])

12,(chemotherapy[Title/Abstract] OR treatment [Title/Abstract] OR therapy[Title/Abstract])

13,11 AND 12

14,5 AND 10 AND 13

**Web of Science**

1,TI=((cervix* OR cervical*OR uterine cervix* OR cervix uteri*)AND(cancer* OR tumor*OR tumour* OR neoplas* OR carcinoma* OR malignanc* OR carcinogenesis* OR intraepithelial neoplas*))

2,TI=((lymph node* OR nodal OR node*OR lymphatic)AND( metastasis OR recurrence OR invasion OR Metastatic Ratio))

3,TI=((neoadjuvant OR preoperat* OR upfront OR primary OR induction OR adjuvant)AND(chemotherapy OR treatment OR therapy))

4,1 AND 2 AND 3

**Cochrane Library**

#1,MeSH descriptor: [Uterine Cervical Neoplasms] explode all trees

#2,cervix* OR cervical*OR uterine cervix* OR cervix uteri*:ti,ab,kw

#3,cancer* OR tumor*OR tumour* OR neoplas* OR carcinoma* OR malignanc* OR carcinogenesis* OR intraepithelial neoplas*:ti,ab,kw

#4,#1 OR (#2 AND #3)

#5,MeSH descriptor: [Lymphatic Metastasis] explode all trees

#6,lymph node* OR nodal or node*OR lymphatic:ti,ab,kw

#7,metastasis OR recurrence OR invasion OR metastatic ratio [Title/Abstract]

#8,#5 OR (#6 AND #7)

#9,neoadjuvant OR preoperat* OR upfront OR primary OR induction OR adjuvant:ti,ab,kw

#10,chemotherapy OR treatment OR therapy:ti,ab,kw

#11,#9 AND #10

#12,#4 AND #8 AND #11

**EMBASE**

1,exp Uterine Cervical Neoplasms/

2,(cervix* or cervical*OR uterine cervix* or cervix uteri*).m_titl.

3,(cancer* or tumor*OR tumour* or neoplas* or carcinoma* or malignanc* or carcinogenesis* or intraepithelial neoplas*).m_titl.

4,2 and 3

5,1 or 4

6,exp Lymphatic Metastasis/

7,(lymph node* or nodal or node*OR lymphatic).m_titl.

8,(metastasis or recurrence or invasion or Metastatic Ratio).m_titl.

9,7 and 8

10,6 or 9

11,(neoadjuvant or preoperat* or upfront or primary or induction or adjuvant).m_titl.

12,(chemotherapy or treatment or therapy).m_titl.

13,11 and 12

14,5 and 10 and 13

Supplementary Table 1. Characteristics of the 34 studies in the meta-analysis.

| Authors | Country | Continent of patients | Study type | Year | Nos. of patients^1^ | Age^2^ | FIGO stage(n,%) | | | | Histological type | | NACT regime | NACT cycle | LNM  (n.%)^3^ |
| --- | --- | --- | --- | --- | --- | --- | --- | --- | --- | --- | --- | --- | --- | --- | --- |
|  |  |  |  |  |  |  | IB1 | IB2 | IIA | IIB | Squamous | Non-  squamous |  |  |  |
| Angioli R et al. | Italy | Europe | prospective study | 2012 | 100 | 48  (29-68) | 0  (0%) | 45  (39%) | 28  (24.4%) | 42  (36.6%) | 87  (75%) | 28  (25%) | Cisplatin + Paclitaxel | 3 cycles | 32  (32%) |
| Cai HB et al. | China | Asia | randomized controlled trial | 2010 | 52 | 45.6±22.4 | 14  (26.9%) | 38  (70.8%) | 0  (0%) | 0  (0%) | 40  (76.9%) | 12  (23.1%) | Cisplatin  +5‐Fu | 2 cycles | 5  (9.6％) |
| Chen H et al. | China | Asia | randomized controlled trial | 2008 | 72 | 44  (25-74) | 0  (0%) | 31  (43.1%) | 21  (29.2%) | 20  (27.8%) | 60  (83.3%) | 12  (16.7%) | cisplatin+  Mitomycin C +5-Fluorouracil | 2-3 cycles | 18  (25%) |
| Cho YH et al. | Korea | Asia | retrospective study | 2009 | 51 | 47.8±10.8 | 0  (0%) | 29  (56.9%) | 22  (43.1%) | 0  (0%) | 46  (90.2%) | 5  (9.8%) | paclitaxel+cisplatin/carboplatin | 2 cycles | 18  (35.3%) |
| Choi CH et al. | Korea | Asia | retrospective study | 2007 | 46 | 46  (29-74) | 0  (0%) | 21  (45.6%) | 10  (21.7%) | 15  (32.6%) | 42  (91%) | 4  (9%) | Vincristine+mitomycin-C+cisplatin | 3 cycles | 16  (34.8%) |
| Costa S et al. | Italy | Europe | retrospective study | 2001 | 21 | 52.5 ± 11.4 | 5  (24%) | 7  (33%) | 9  (43%) | 0  (0%) | 17  (81%) | 4  (19%) | cisplatin+ epirubicin+etoposide+bleomycin | 3 cycles | 3  (14%) |
| Eddy GL et al. | USA | ^4^ | randomized controlled trial | 2007 | 145 | ≤30:14%,31-40:31%,41-50:32%,51-60:15%,≥61:7% | 145(100%) | | 0  (0%) | 0  (0%) | 113  (78%) | 22  (15%) | vincristine+cisplatin | 3 cycles | pelvic:47 (32.4%) |
| Gadducci A et al. | Italy | Europe | retrospective study | 2010 | 140 | 47  (22-79) | 0  (0%) | 48  (34.3%) | 32  (22.9%) | 60  (42.8%) | 120  (85.7%) | 20  (14.3%) | TIP,TP,PVB,TEP,IP,P+5-FU | 3 cycles | 33  (23.6%) |
| Gong L et al. | China | Asia | retrospective study | 2012 | 202 | 43.1±8.0 | 0  (0%) | 33  (16%) | 54  (27%) | 115  (57%) | 180  (89%) | 22  (11%) | BP,5-Fu+BP,FIP,TP/TC,BVP | 1–3 cycles | 56  (27.7%) |
| Gupta S et al. | India | Asia | randomized controlled trial | 2018 | 316 | 50  (27-65) | 0  (0%) | 57  (18%) | 80  (25.3%) | 179  (56.7%) | 316  (100%) | 0  (0%) | paclitaxel+carboplatin | 3 cycles | pelvic:46  (14.6%) |
| Hu T et al. | China | Asia | retrospective study | 2015 | 705 | 43.7±8.9 | 126  (17.9%) | 204  (28.9%) | 247  (35.1%) | 128  (18.2%) | 638  (90.5%) | 67  (9.5%) | cisplatin-  based chemotherapy | NA | 140  (19.9%) |
| Huang X et al. | China | Asia | retrospective study | 2011 | 52 | 46  (30-63) | 0  (0%) | 23  (44.2%) | 11  (21.2%) | 18  (34.8%) | 44  (84.6%) | 8  (15.4%) | docetaxel+cisplatin | 2-3 cycles | 7  (13.7%) |
| Hwang YY et al. | Korea | Asia | retrospective study | 2001 | 80 | 47.5  (27-64) | 11(13.8%) | | 39  (48.8%) | 30  (37.5%) | 73  (91.3%) | 7  (8.7%) | VBP | 1-6 cycles | 17  (21.3%) |
| Katsumata N et al. | Japan | Asia | randomised controlled trial | 2013 | 67 | 47(28-70) | 0  (0%) | 24  (36%) | 5  (8%) | 38  (57%) | 67  (100%) | 0(0%) | BOMP( bleomycin+vincristine+mitomycin+cisplatin) | 2-4 | pelvic:17  (27%)^5^ |
| Kim HS et al. | Korea | Asia | retrospective study | 2011 | 73 | 49.4±9.8 | 93  (20.6%) | 11  (15.1%) | 39  (53.4%) | 0  (0%) | 63  (86.3%) | 10  (13.7%) | 5-FU/  cisplatin,  Paclitaxel/  carboplatin | 1-4 cycles | 17  (23.3%) |
| Lee JY et al. | Korea | Asia | retrospective study | 2011 | 33 | 47.5±9.6 | 0  (0%) | 14  (42.4%) | 19  (57.6%) | 0  (0%) | 27  (81.8%) | 6  (18.2%) | paclitaxel+carboplatin,5 FU+cisplatin | 2-3 cycles | 4  (12.1%) |
| Li D et al. | China | Asia | retrospective study | 2012 | 104 | NA | 0  (0%) | 56  (53.8%) | 22  (21.2%) | 26  (25%) | 89  (85.6%) | 15  (14.4%) | carboplatin+taxol | 1-3 cycles | 21  (20.2%) |
| Li R et al. | China | Asia | retrospective study | 2013 | 154 | 41.6±7.92 | 0  (0%) | 74  (48.1%) | 80  (51.9%) | 0  (0%) | 147  (95.55) | 7  (4.5%) | TP,BVP | 1-2 cycles | 20  (13%) |
| Lorusso d et al. | Italy | Europe | retrospective study | 2014 | 30 | 45.7  (15-75) | 0  (0%) | 9  (30%) | 0  (0%) | 21  (70%) | 0  (0%) | 30  (100%) | TAP | 3 cycles | 10  (33.3%) |
| Martinelli F et al. | Italy | Europe | retrospective study | 2015 | 275 | 44  (18-77) | 0  (0%) | 107  (39%) | 51  (19%) | 117  (42%) | 221  (80%) | 54  (20%) | platinum + paclitaxel+ topotecan+  adryamicin or ifosfamide+ irinotecan+ bleomycin | 2-4 cycles | 58  (21.1%) |
| Namkoong SE et al. | Korea | Asia | retrospective study | 1995 | 92 | 48  (30-58) | 41(44.5%) | | 36  (39.1%) | 15  (16.3%) | 84  (91%) | 8  (9%) | vinblastin+bleomycin+cisplatin | 2-5 cycles | 16  (17.4%) |
| Prueksaritanond N et al. | Thailand | Asia | retrospective study | 2012 | 40 | 42.98 ±6.67) | 0  (0%) | 27 (67.5%) | 13 (32.5%) | 0  (0%) | 34 (85.0%) | 6 (15.0%) | paclitaxel+carboplatin | 1-3 cycles | 9  (22.5%) |
| Robova H et al. | Czech Republic | Europe | prospective study | 2010 | 132 | NA | 30 (21.3%) | 111  (78.7%) | 0  (0%) | 0  (0%) | 125  (88.7%) | 16  (11.3%) | Cisplatin+ifosfamide,Cisplatin+doxorubicin | 3 cycles | 22  (16.7%) |
| Sardi JE et al. | Argentina | South America and North America | randomized controlled trial | 1997 | 98 | 38.5  (24-63) | 37  (37.8%) | 61  (62.2%) | 0  (0%) | 0  (0%) | 98  (100%) | 0  (0%) | VBP | 3 cycles | 8  (8.2%) |
| Serur E et al. | USA | South America and North America | retrospective study | 1997 | 20 | 47.8 ± 13.7 | 0  (0%) | 20  (100%) | 0  (0%) | 0  (0%) | 20  (100%) | 0  (0%) | cisplatin+ methotrexate+ bleomycin,cisplatin+ vincristine+bleomycin | 3 cycles | 2  (10%) |
| Takatori E et al. | Japan | Asia | retrospective study | 2015 | 33 | 42  (25-63) | 0  (0%) | 8  (24.2%) | 2  (6.1%) | 23  (69.7%) | 33  (100%) | 0  (0%) | CDDP + CPT-11 | 2 cycles | 11  (33.3%) |
| Vizza E et al. | Italy | Europe | retrospective study | 2014 | 60 | 44  (19-72) | 0  (0%) | 34  (56.6%) | 7  (11.7%) | 19  (31.7%) | 40  (66.7%) | 20  (33.3%) | TIP,TEP,  other Platinum based regime | 3 cycles | 13  (21.7%) |
| Watari H et al. | Japan | Asia | retrospective study | 2010 | 46 | 46.5  (27-64) | 0  (0%) | 6  (13%) | 4  (8.7%) | 36  (78.3%) | 25  (54.3%) | 21  (45.7%) | BOMP,ITP,TP,MEP,CAP,,TC, DC,,CDDP | 1-4 cycles | 20  (43.5%) |
| Wen H et al. | China | Asia | randomized controlled trial | 2012 | 28 | 44.53±9.10 | 0(0%) | 8  (26.7%) | 22 (73.3%) | 0  (0%) | 28 (93.3%) | 2  (6.7%) | cisplatin+5-Fluorouracil | 2 cycles | 5 (17.9%) |
| Xie Q et al. | China | Asia | retrospective study | 2015 | 52 | 43  (27-63) | 0  (0%) | 28  (53.8%) | 24  (46.2%) | | 42  (80.8%) | 10  (19.2%) | TP,PF | 2-3 cycles | 25  (48.1%) |
| Yang Z et al. | China | Asia | randomized controlled trial | 2016 | 109 | 47  (23-66) | 0  (0%) | 19  (17.4%) | 18  (16.5%) | 72  (66.1%) | 91  (83.5%) | 18  (16.5%) | IP,TP | 1-2 cycles | 22  (20.6%) |
| Yin M et al. | China | Asia | retrospective study | 2011 | 187 | 43  (26-68) | 0  (0%) | 31  (16.6%) | 78  (41.7%) | 78  (41.7%) | 172  (92.0%) | 15  (8.0%) | TP,PVB | 2-3 cycles | 43  (23%) |
| Zanaboni F et al. | Italy | Europe | prospective study | 2013 | 81 | 48  (24-74) | 0  (0%) | 30  (33％) | 13  (14％) | 49  (53％) | 89  (97％) | 3  (3%) | topotecan+cisplatin | 6 cycles | 30  (37%) |
| Zhang Y et al. | China | Asia | retrospective study | 2019 | 117 | ＜45:43.6%,≥45:56.4% | 0  (0%) | 53  (45.3%) | 64  (54.7%) | 0  (0%) | 117  (100%) | 0  (0%) | paclitaxel+ carboplatin | 3 cycles | 56  (47.9%) |

^1^The number of patients who received the neoadjuvant chemotherapy plus radical hysterectomy.

^2^As for the information about age,some studies offered medium and range,some studies offered the mean and standard deviation,and others provide the different age ranges and corresponding patient proportions

^3^The number of patients who had lymph node metastases and the proportion.

^4^The patients in this study consisted of:62% were white,13% were black,19% were Hispanic,4% were Asian/Pacific islander and 1% were Other.

^5^ In this study, there are 67 patients in two group, and in each group, 62 (93%) underwent RS, also suggesting that operability was similar in the groups.

Supplementary Table 2. The definition of risk of bias.

| Domain | Low risk of bias | Unclear risk of bias | High risk of bias |
| --- | --- | --- | --- |
| Assessment of record | Reliable records (e.g. hospital or institute records,institutional review board approval data) | Insufficient information about the assessment of record to permit judgment of "Low risk" or "High risk" | Unreliable records (e.g.structured interview, self-written report records) |
| Assessment of diagnosis | Histological diagnosis | Insufficient information about the assessment of diagnosis to permit judgment of "Low risk" or "High risk" | Diagnosis by schiller test,endocervical scraping smear examination and/or colposcopy |
| Assessment of LN status | LN status confirmed with the surgical findings | Insufficient information about the assessment of LN status to permit judgment of "Low risk" or "High risk" | LN status confirmed with the other methods(e.g.imaging) |
| Assessment of loss to follow-up^1^ | Loss to follow-up less than 5% | Insufficient information about the assessment of follow-up to permit judgment of "Low risk" or "High risk" | Loss to follow-up over 10% or not adequately reported |
| Selective inclusion and exclusion | Avoiding selective and inappropriate inclusion and exclusion | Insufficient information about the selective reporting to permit judgment of "Low risk" or "High risk" | Selective and inappropriate inclusion and exclusion |
| ^1^The loss to follow-up rate was defined as the rate of the number of women lost to follow-up in prospective studies or patients with missing data in retrospective cohort studies divided by the number of initial total patients | | | |

Supplementary Table 3. The assessment of risk of bias.

| Author | Year of publication | Assessment of record | Assessment of diagnosis | Assessment of LN status | Assessment of follow-up | Selective inclusion and exclusion | summary bias risk^1^ |
| --- | --- | --- | --- | --- | --- | --- | --- |
| Angioli R et al. | 2012 | Low risk of bias | Low risk of bias | Low risk of bias | High risk of bias | Low risk of bias | High risk of bias |
| Cai HB et al. | 2010 | Low risk of bias | Low risk of bias | Low risk of bias | Low risk of bias | Low risk of bias | Low risk of bias |
| Chen H et al. | 2008 | Low risk of bias | Low risk of bias | Low risk of bias | Low risk of bias | Low risk of bias | Low risk of bias |
| Cho YH et al. | 2009 | Unclear risk of bias | Low risk of bias | Low risk of bias | Low risk of bias | Low risk of bias | High risk of bias |
| Choi CH et al. | 2007 | Low risk of bias | Low risk of bias | Low risk of bias | Low risk of bias | Low risk of bias | Low risk of bias |
| Costa S et al. | 2001 | Low risk of bias | Low risk of bias | Low risk of bias | High risk of bias | Low risk of bias | High risk of bias |
| Eddy GL et al. | 2007 | Low risk of bias | Low risk of bias | High risk of bias | Low risk of bias | Low risk of bias | High risk of bias |
| Gadducci A et al. | 2010 | Low risk of bias | Low risk of bias | Low risk of bias | Low risk of bias | Low risk of bias | Low risk of bias |
| Gong L et al. | 2012 | Low risk of bias | Low risk of bias | Low risk of bias | Low risk of bias | Low risk of bias | Low risk of bias |
| Gupta S et al. | 2018 | Low risk of bias | Low risk of bias | High risk of bias | Low risk of bias | Low risk of bias | High risk of bias |
| Hu T et al. | 2015 | Low risk of bias | Low risk of bias | Low risk of bias | Low risk of bias | Low risk of bias | Low risk of bias |
| Huang X et al. | 2011 | Low risk of bias | Low risk of bias | Low risk of bias | Low risk of bias | Low risk of bias | Low risk of bias |
| Hwang YY et al. | 2001 | Low risk of bias | Low risk of bias | Low risk of bias | Low risk of bias | Low risk of bias | Low risk of bias |
| Katsumata N et al. | 2013 | Low risk of bias | Low risk of bias | High risk of bias | Low risk of bias | Low risk of bias | High risk of bias |
| Kim HS et al. | 2011 | Low risk of bias | Low risk of bias | Low risk of bias | Low risk of bias | Low risk of bias | Low risk of bias |
| Lee JY et al. | 2011 | Low risk of bias | Low risk of bias | Low risk of bias | Low risk of bias | Low risk of bias | Low risk of bias |
| Li D et al. | 2012 | Low risk of bias | Low risk of bias | Low risk of bias | Low risk of bias | Low risk of bias | Low risk of bias |
| Li R et al. | 2013 | Low risk of bias | Low risk of bias | Low risk of bias | Low risk of bias | Low risk of bias | Low risk of bias |
| Lorusso d et al. | 2014 | Low risk of bias | Low risk of bias | Low risk of bias | Low risk of bias | Low risk of bias | Low risk of bias |
| Martinelli F et al. | 2015 | Low risk of bias | Low risk of bias | Low risk of bias | Low risk of bias | Low risk of bias | Low risk of bias |
| Namkoong SE et al. | 1995 | Low risk of bias | Low risk of bias | Low risk of bias | Low risk of bias | Low risk of bias | Low risk of bias |
| Prueksaritanond N et al. | 2012 | Low risk of bias | Low risk of bias | Low risk of bias | Low risk of bias | Low risk of bias | Low risk of bias |
| Robova H et al. | 2010 | Low risk of bias | Low risk of bias | Low risk of bias | Unclear risk of bias | Low risk of bias | High risk of bias |
| Sardi JE et al. | 1997 | Low risk of bias | Low risk of bias | Low risk of bias | Low risk of bias | Low risk of bias | Low risk of bias |
| Serur E et al. | 1997 | Low risk of bias | Low risk of bias | Unclear risk of bias | Low risk of bias | Low risk of bias | High risk of bias |
| Takatori E et al. | 2015 | Low risk of bias | Low risk of bias | Low risk of bias | Low risk of bias | Low risk of bias | Low risk of bias |
| Vizza E et al. | 2014 | Low risk of bias | Low risk of bias | Low risk of bias | Low risk of bias | Low risk of bias | Low risk of bias |
| Watari H et al. | 2010 | Low risk of bias | Low risk of bias | Low risk of bias | Low risk of bias | Low risk of bias | Low risk of bias |
| Wen H et al. | 2012 | Low risk of bias | Low risk of bias | Low risk of bias | Unclear risk of bias | Low risk of bias | High risk of bias |
| Xie Q et al. | 2015 | Low risk of bias | Low risk of bias | Low risk of bias | Low risk of bias | Low risk of bias | Low risk of bias |
| Yang Z et al. | 2016 | Low risk of bias | Low risk of bias | Low risk of bias | Low risk of bias | Low risk of bias | Low risk of bias |
| Yin M et al. | 2011 | Low risk of bias | Low risk of bias | Low risk of bias | Low risk of bias | Low risk of bias | Low risk of bias |
| Zanaboni F et al. | 2013 | Low risk of bias | Low risk of bias | Low risk of bias | High risk of bias | Low risk of bias | High risk of bias |
| Zhang Y et al. | 2019 | Low risk of bias | Low risk of bias | Low risk of bias | Low risk of bias | Low risk of bias | Low risk of bias |

Supplementary Table 4. Freeman-Tukey double arcsine transformation.

| Author | Year of publication | Nos. of patients^1^ | E^2^ | R^3^ | Transformed-ES^4^ | 95% Conf. Interval | Weight % |
| --- | --- | --- | --- | --- | --- | --- | --- |
| Angioli R et al. | 2012 | 100 | 32 | 0.32 | 1.206 | 1.011-1.401 | 3.2 |
| Cai HB et al. | 2010 | 52 | 5 | 0.096 | 0.655 | 0.386-0.925 | 2.6 |
| Chen H et al. | 2008 | 72 | 18 | 0.25 | 1.055 | 0,826-1.284 | 2.9 |
| Cho YH et al. | 2009 | 51 | 18 | 0.353 | 1.278 | 1.006-1.550 | 2.6 |
| Choi CH et al. | 2007 | 46 | 16 | 0.348 | 1.268 | 0.982-1.554 | 2.5 |
| Costa S et al. | 2001 | 21 | 3 | 0.14 | 0.819 | 0.401-1.237 | 1.7 |
| Eddy GL et al. | 2007 | 145 | 47 | 0.324 | 1.214 | 1.052-1.376 | 3.4 |
| Gadducci A et al. | 2010 | 140 | 33 | 0.236 | 1.018 | 0.853-1.183 | 3.4 |
| Gong L et al. | 2012 | 202 | 56 | 0.277 | 1.111 | 0.974-1.249 | 3.6 |
| Gupta S et al. | 2018 | 316 | 46 | 0.146 | 0.786 | 0.676-0.896 | 3.8 |
| Hu T et al. | 2015 | 705 | 140 | 0.199 | 0.925 | 0.851-0.999 | 3.9 |
| Huang X et al. | 2011 | 52 | 7 | 0.137 | 0.771 | 0.502-1.040 | 2.6 |
| Hwang YY et al. | 2001 | 80 | 17 | 0.213 | 0.967 | 0.749-1.185 | 3.0 |
| Katsumata N et al. | 2013 | 67 | 17 | 0.27 | 1.064 | 0.826-1.302 | 2.9 |
| Kim HS et al. | 2011 | 73 | 17 | 0.233 | 1.016 | 0.788-1.243 | 2.9 |
| Lee JY et al. | 2011 | 33 | 4 | 0.121 | 0.744 | 0.408-1.080 | 2.2 |
| Li D et al. | 2012 | 104 | 21 | 0.202 | 0.939 | 0.748-1.130 | 3.2 |
| Li R et al. | 2013 | 154 | 20 | 0.13 | 0.744 | 0.587-0.902 | 3.4 |
| Lorusso d et al. | 2014 | 30 | 10 | 0.333 | 1.242 | 0.890-1.594 | 2.1 |
| Martinelli F et al. | 2015 | 275 | 58 | 0.211 | 0.957 | 0.839-1.075 | 3.7 |
| Namkoong SE et al. | 1995 | 92 | 16 | 0.174 | 0.869 | 0.666-1.073 | 3.1 |
| Prueksaritanond N et al. | 2012 | 40 | 9 | 0.225 | 1.004 | 0.698-1.310 | 2.4 |
| Robova H et al. | 2010 | 132 | 22 | 0.167 | 0.848 | 0.678-1.018 | 3.4 |
| Sardi JE et al. | 1997 | 98 | 8 | 0.082 | 0.595 | 0.398-0.792 | 3.2 |
| Serur E et al. | 1997 | 20 | 2 | 0.1 | 0.701 | 0.274-1.129 | 1.7 |
| Takatori E et al. | 2015 | 33 | 11 | 0.333 | 1.241 | 0.905-1.577 | 2.2 |
| Vizza E et al. | 2014 | 60 | 13 | 0.217 | 0.979 | 0.728-1.230 | 2.8 |
| Watari H et al. | 2010 | 46 | 20 | 0.435 | 1.443 | 1.157-1.729 | 2.5 |
| Wen H et al. | 2012 | 28 | 5 | 0.179 | 0.900 | 0.536-1.264 | 2.0 |
| Xie Q et al. | 2015 | 52 | 25 | 0.481 | 1.533 | 1.264-1.802 | 2.6 |
| Yang Z et al. | 2016 | 109 | 22 | 0.206 | 0.939 | 0.752-1.125 | 3.2 |
| Yin M et al. | 2011 | 187 | 43 | 0.23 | 1.004 | 0.861-1.147 | 3.5 |
| Zanaboni F et al. | 2013 | 81 | 30 | 0.37 | 1.312 | 1.095-1.528 | 3.0 |
| Zhang Y et al. | 2019 | 117 | 56 | 0.479 | 1.528 | 1.348-1.709 | 3.3 |
| transformed ES：1.02，95%CI:0.95-1.09 | | | | | | | |
| pooled ES:0.238,95%CI:0.209-0.269 | | | | | | | |

1The numbers of the total patients included in each study.

2The numbers of the patients who have positive lymph nodes in each group.

3The ratios of the patients who have positive lymph nodes in each group.

4The estimates after the Freeman-Tukey double arcsine transformation.

Supplementary Table 5 Characteristics of the 5 RCTs reporting the 5-year OS in the meta-analysis.

| Author | Country | Year of publication | Nos. of total patients | Age(medium,range)or  (mean,SD) | Patients in NACT+surgery group | | Patients in surgery group | |
| --- | --- | --- | --- | --- | --- | --- | --- | --- |
|  |  |  |  |  | Nos. of OS | Nos. of Total | Nos. of OS | Nos. of Total |
| Cai HB et al. | China | 2010 | 106 | 45.6±22.4 | 44 | 52 | 41 | 54 |
| Chen H et al. | China | 2008 | 142 | 44（25-74） | 51 | 72 | 41 | 70 |
| Eddy GL et al. | USA | 2007 | 288 | ≤30:14%,31-40:31%,41-50:32%,51-60:15%,≥61:7% | 92 | 145 | 87 | 143 |
| Sardi JE et al. | Argentina | 1997 | 201 | 38.5(24-63) | 82 | 98 | 73 | 103 |
| Wen H et al. | China | 2012 | 60 | 44.53±9.10 | 23 | 28 | 23 | 32 |

Supplementary Figure. 1.

The sensitivity analyses did not noticeably affect the results

Supplementary Figure. 2.

the visual inspection of funnel plots showed no evidence of the presence of small study effects

Supplementary Figure. 3. the Egger’s test

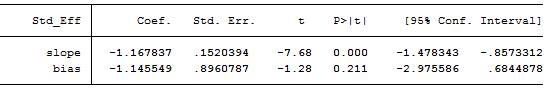


When P >I t I, the p value was 0.211(>0.1). That suggested no obvious bias of publication.

Supplementary Figure. 4A


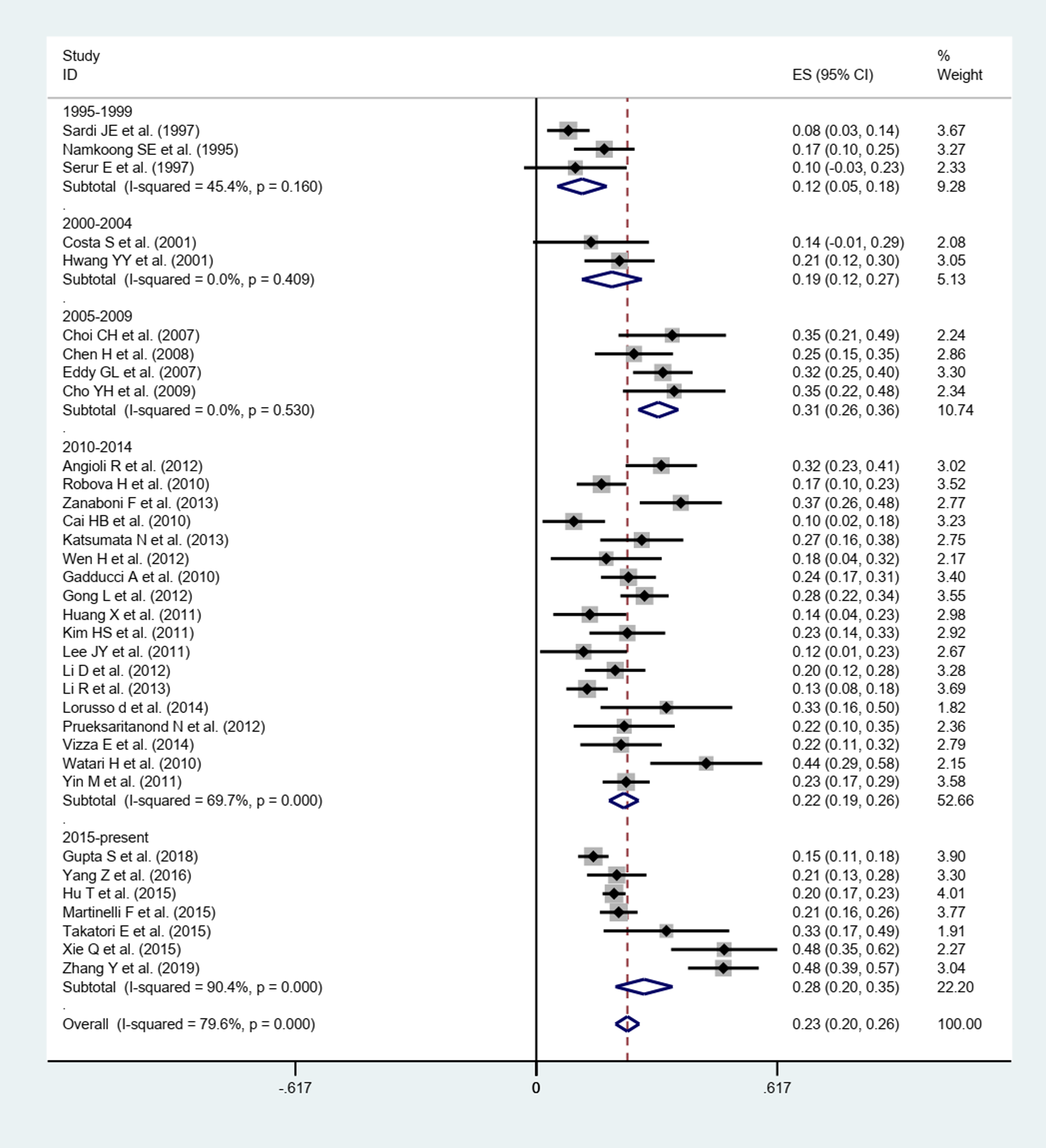


Supplementary Figure. 4A showed the rate of positive lymph nodes in subgroups defined by the year of publication.Supplementary Figure. 4B


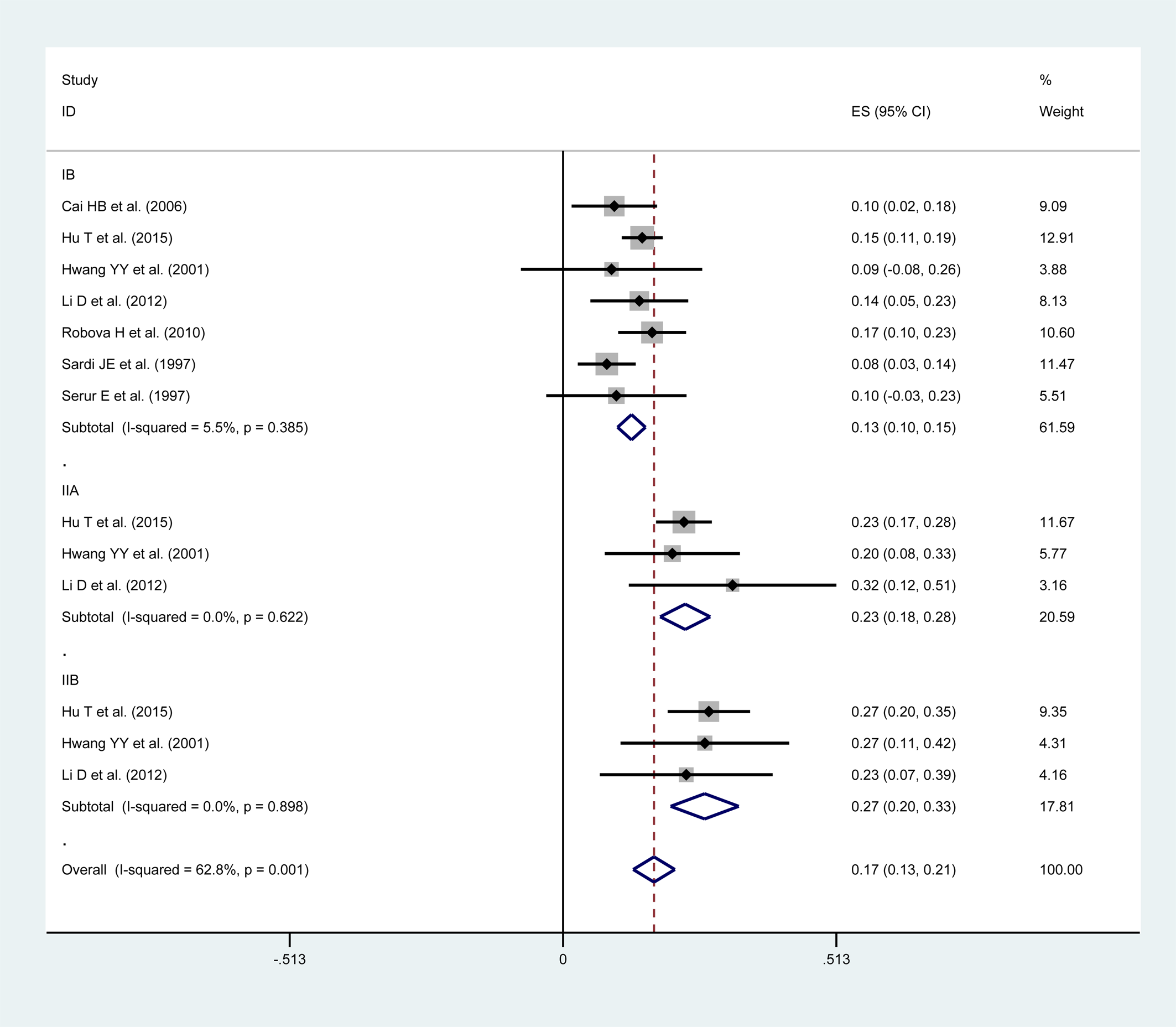


Supplementary Figure. 4B showed the rate of positive lymph nodes in subgroups defined by the FIGO stage.

Supplementary Figure 5


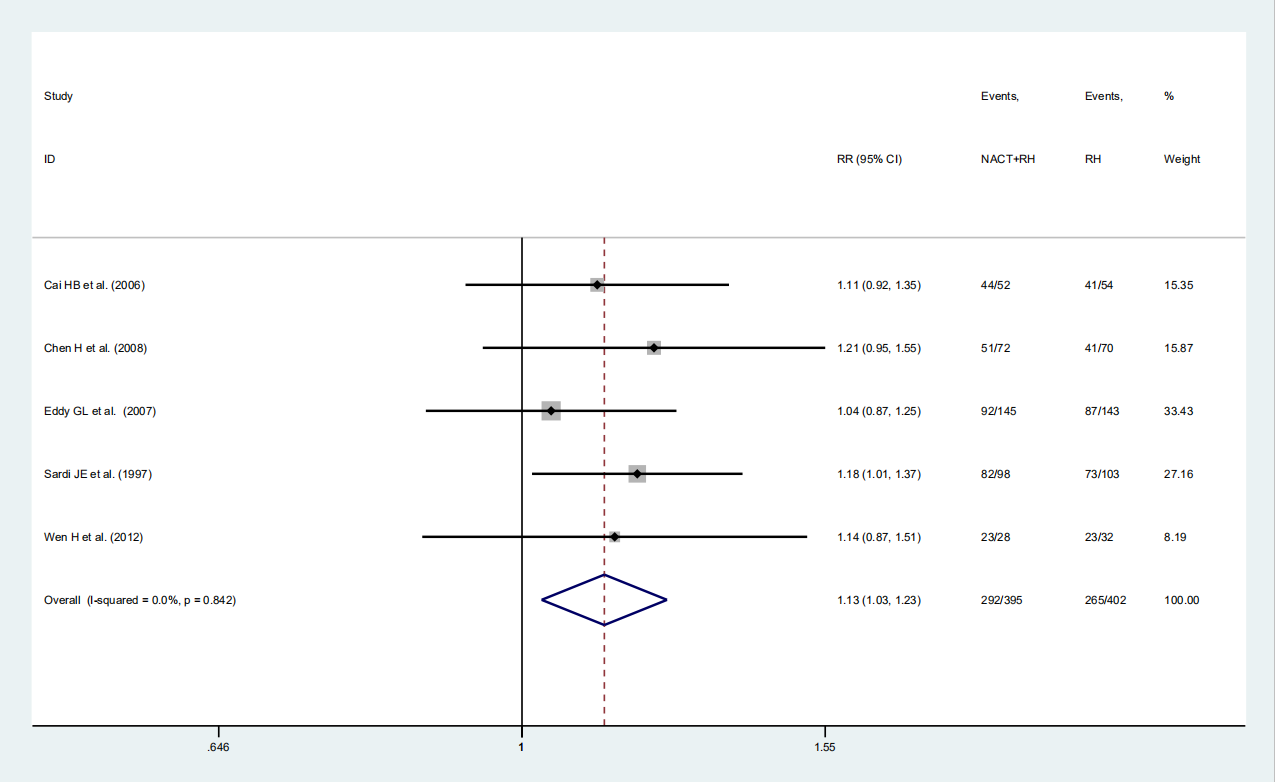


Supplementary Figure 5 Forest plots for the 5-year OS in the comparison between NACT plus RH and RH.

Supplementary Figure. 6


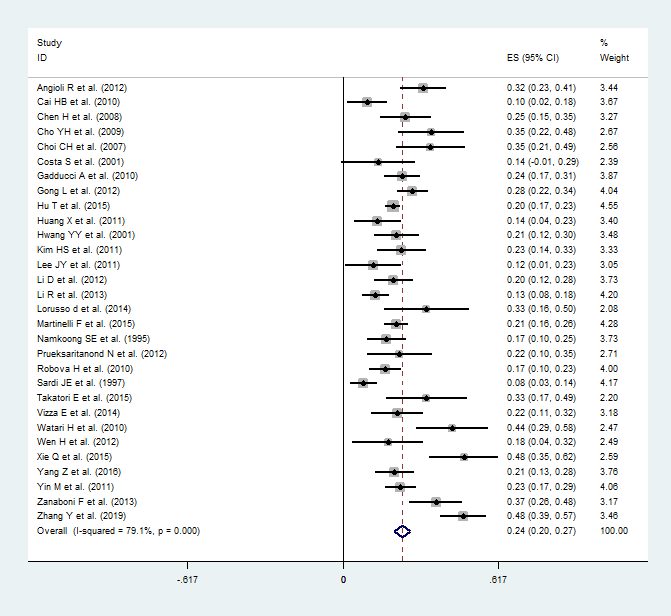


Supplementary Figure. 6. Meta-analysis in the 30 studies that determined para-aortic lymph nodes and pelvic lymph nodes by histological biopsy.
