# Supplementary figures and images for: The Effect of Neoadjuvant Chemotherapy on Lymph Node Metastasis of FIGO Stage IB1-IIB Cervical Cancer: A Systematic Review and Meta-Analysis
Source: Front Oncol. 2020 Nov 5;10:570258. doi: 10.3389/fonc.2020.570258 (PMC7675063; doi:10.3389/fonc.2020.570258)

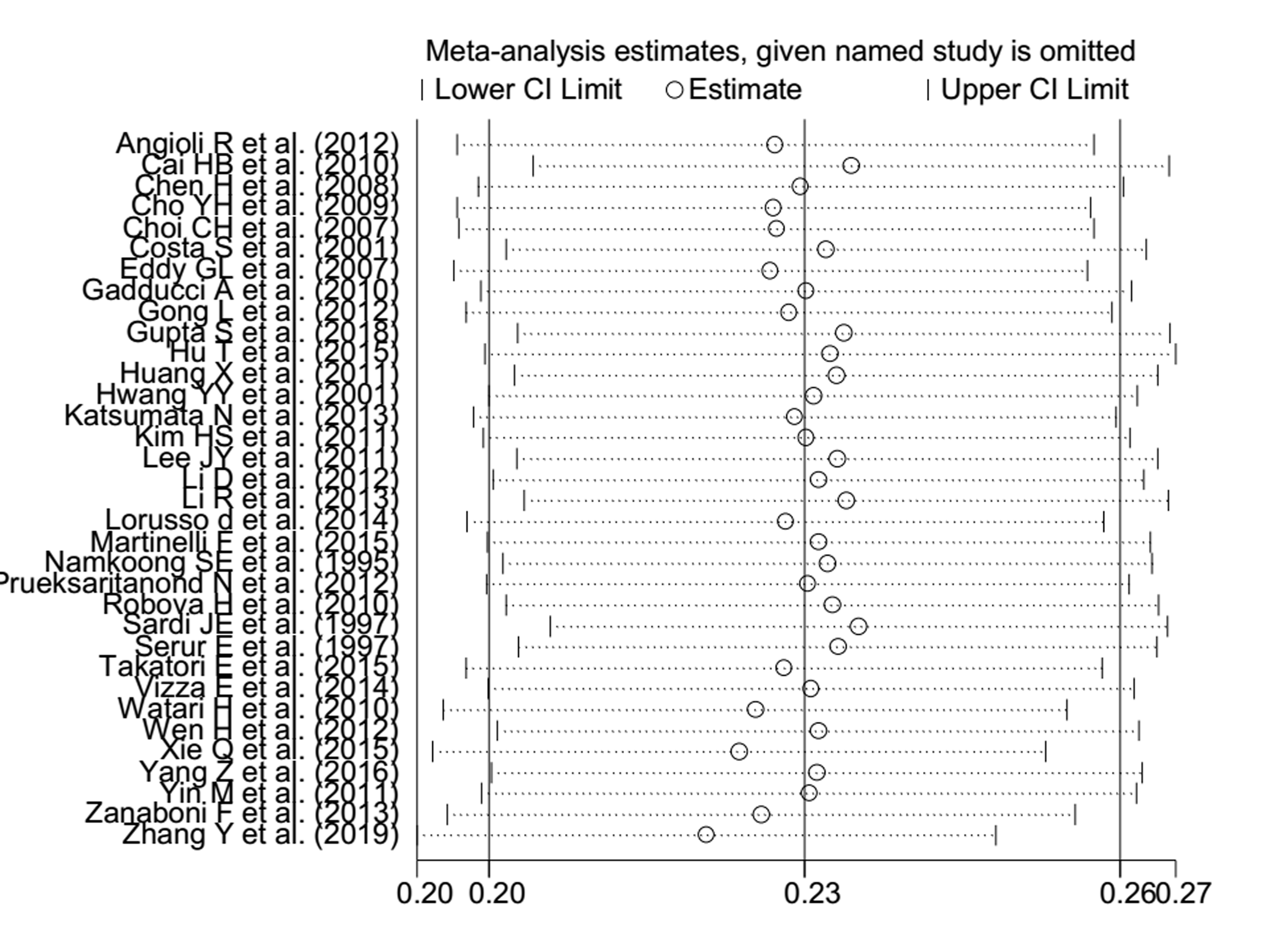

Supplement: Supplementary file 2 [file Image_1.tif]

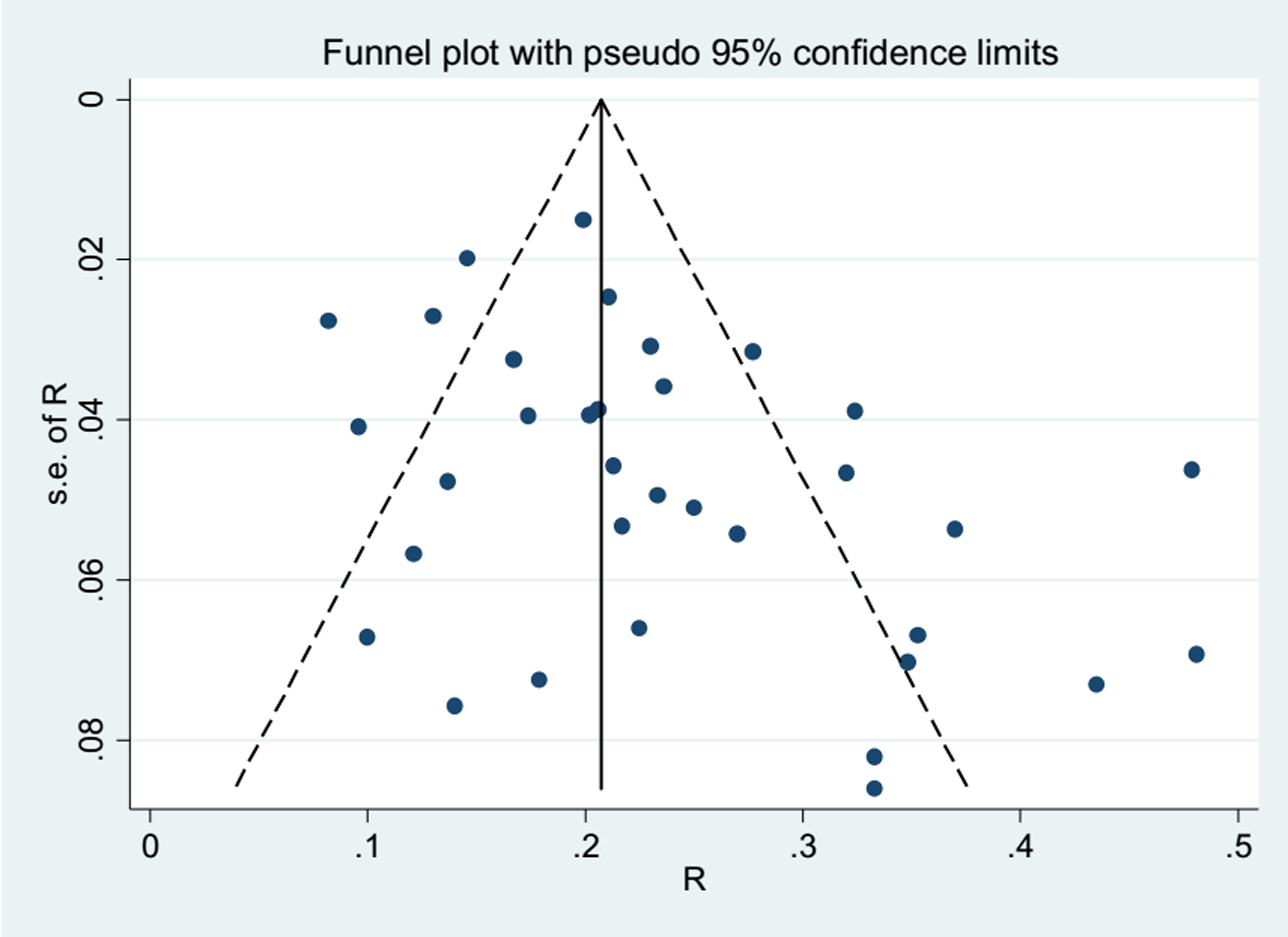

Supplement: Supplementary file 3 [file Image_2.tif]

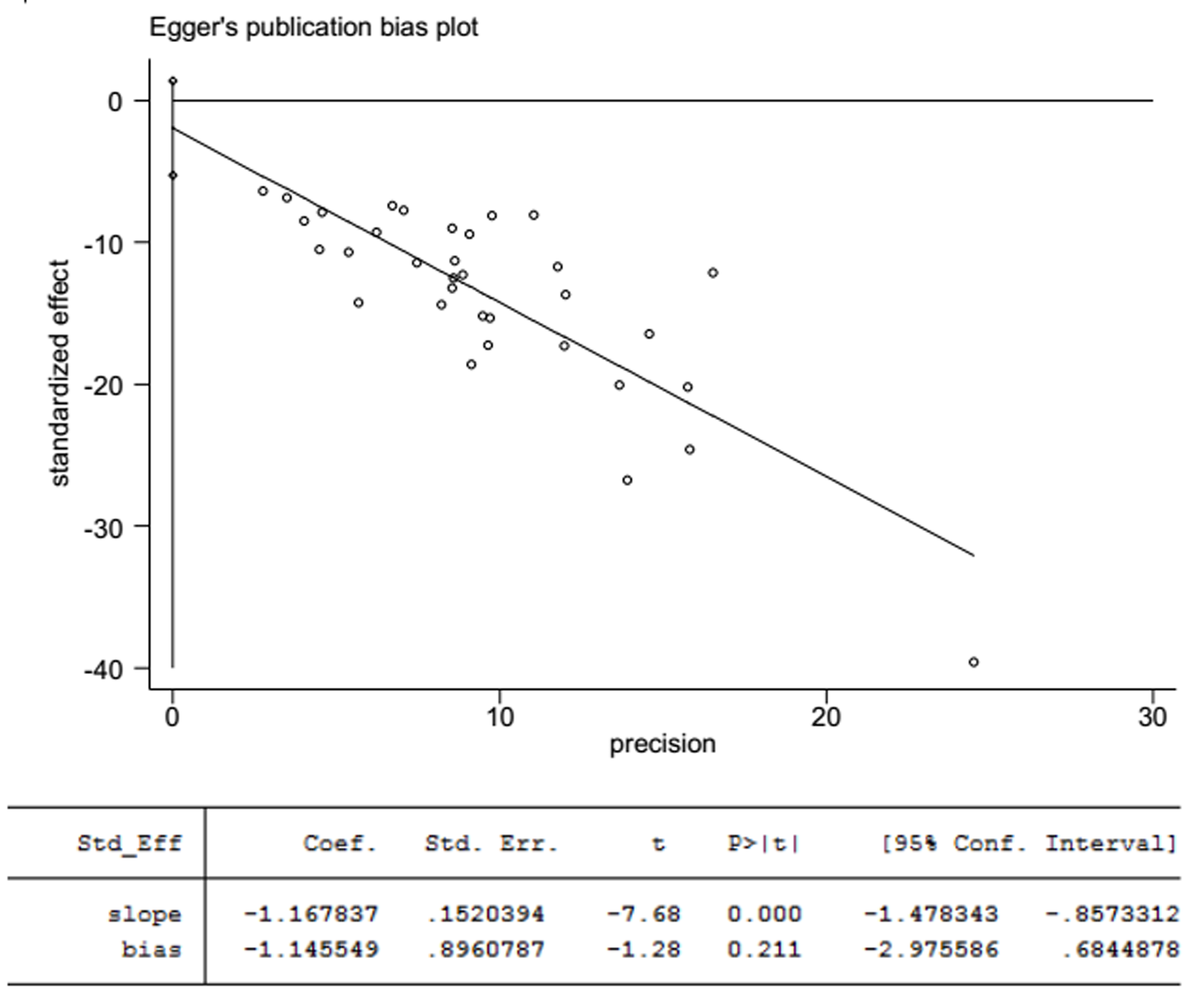

Supplement: Supplementary file 4 [file Image_3.tif]

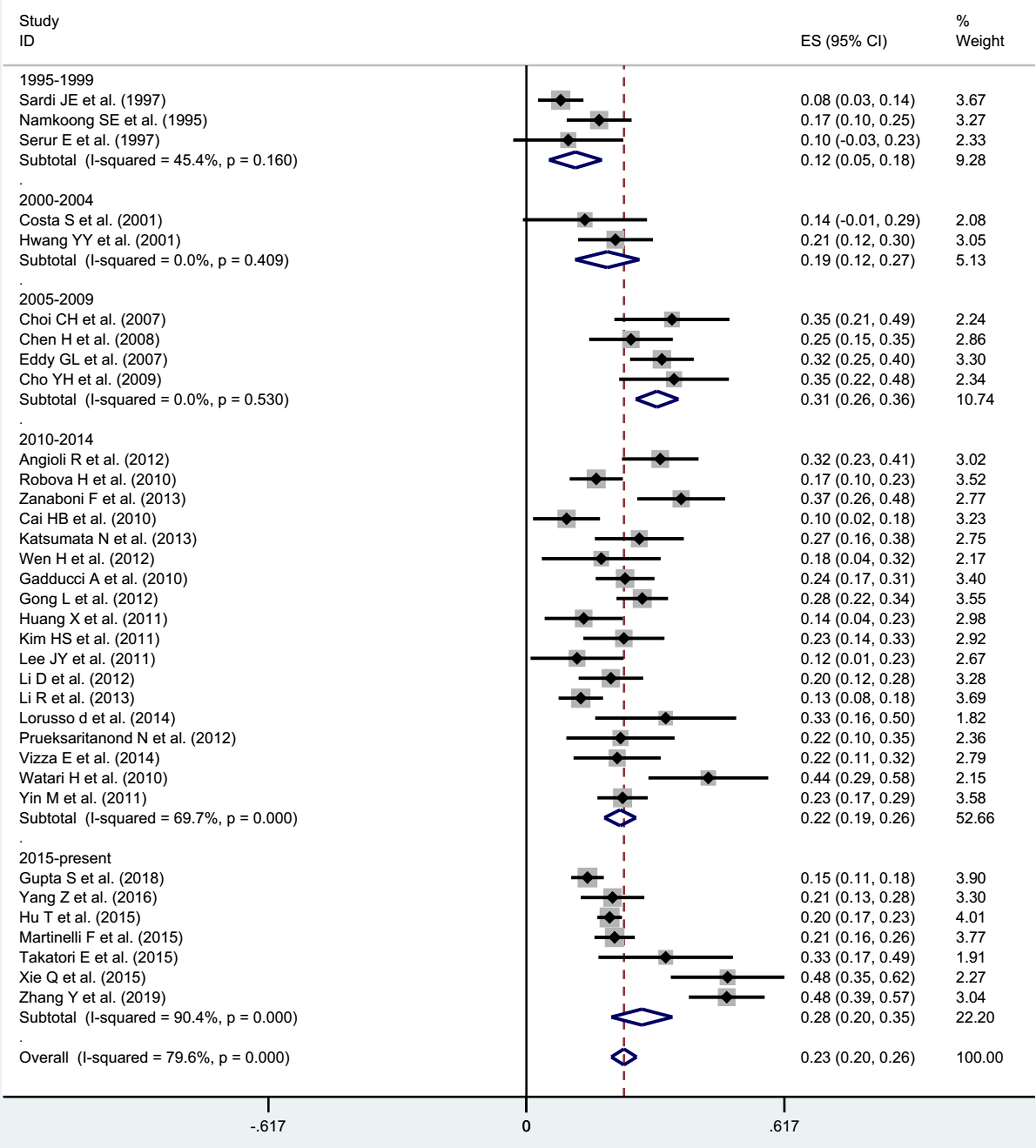

Supplement: Supplementary file 5 [file Image_4.tif]

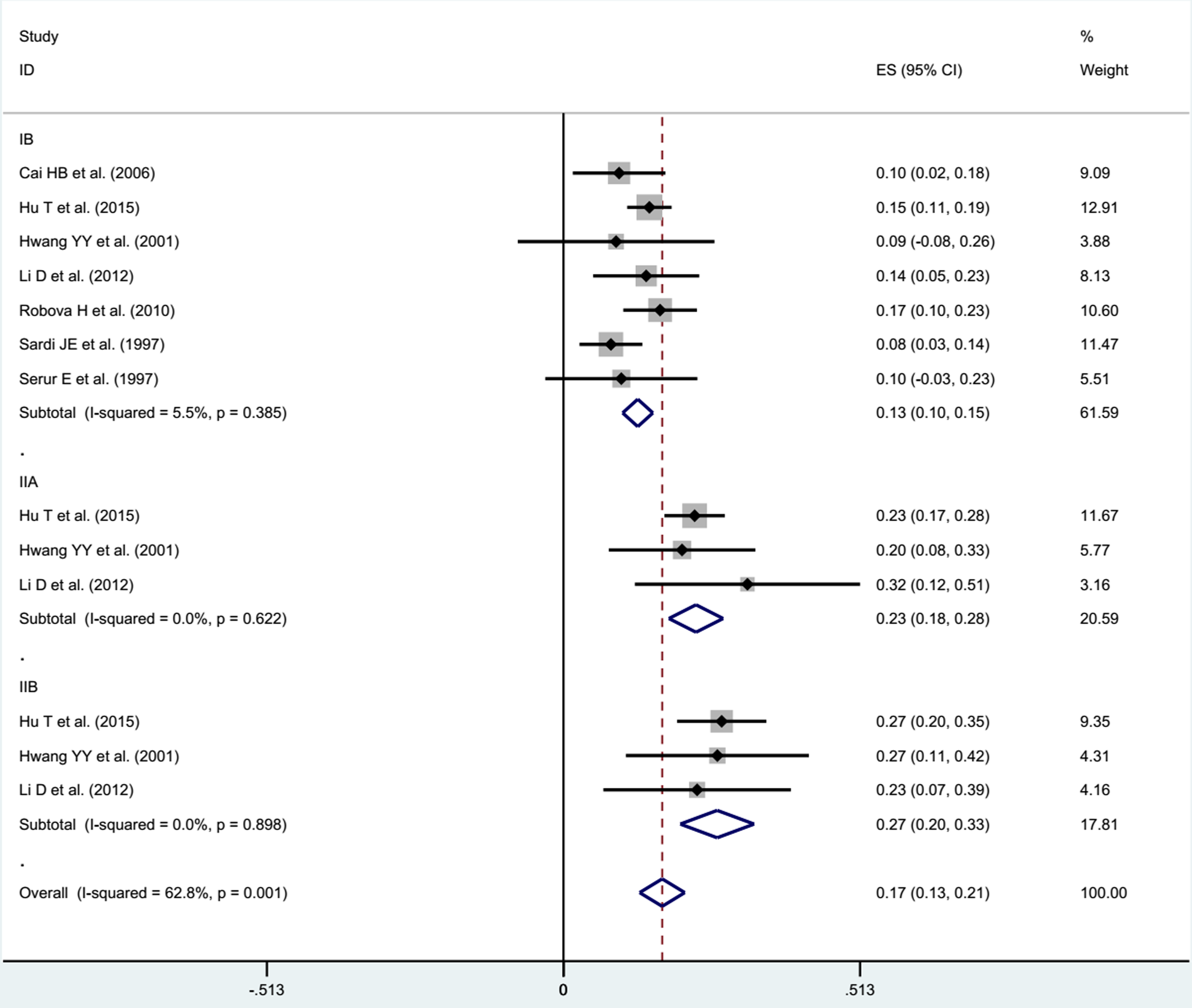

Supplement: Supplementary file 6 [file Image_5.tif]

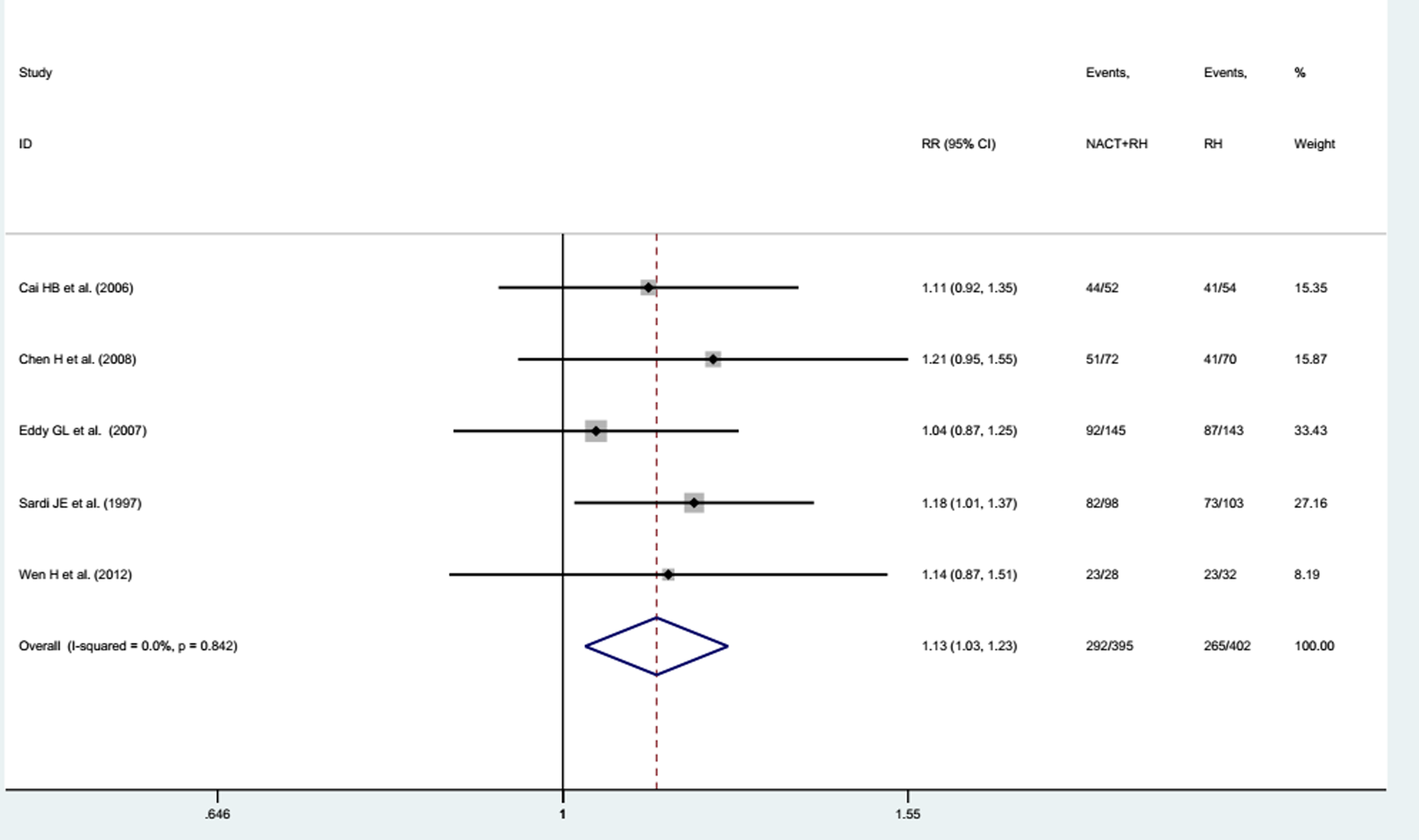

Supplement: Supplementary file 7 [file Image_6.tif]

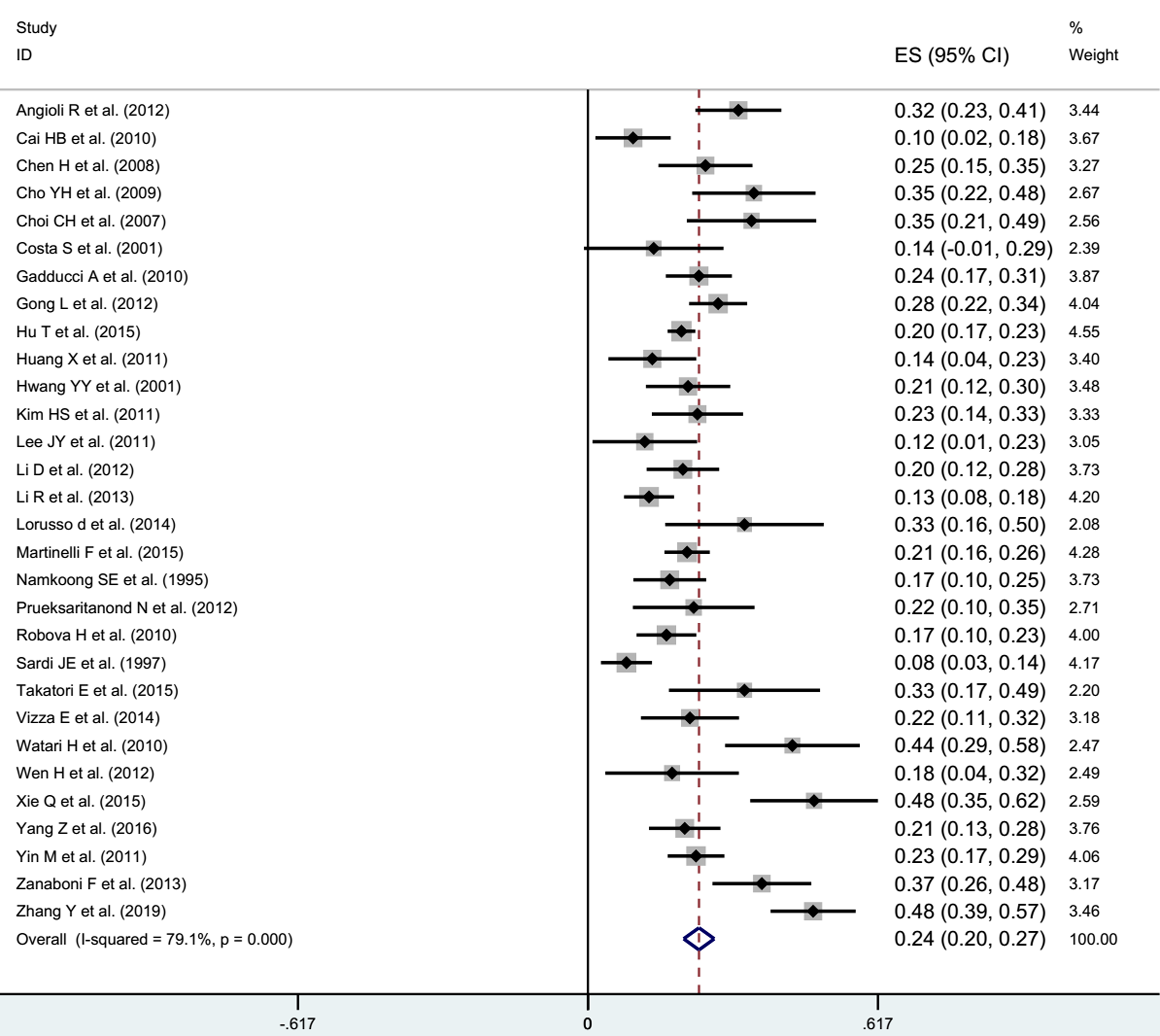

Supplement: Supplementary file 8 [file Image_7.tif]
